# hnRNPA1-SF3B3 interaction drives radioresistance in oral squamous cell carcinoma by modulating MARF1 alternative splicing isoforms

**DOI:** 10.1186/s13046-026-03697-4

**Published:** 2026-03-21

**Authors:** Fan-tong Xia, Si-qi Cai, Jie-ying Yang, Jia Jiang, Yi-hong Hu, Huan Zhang, Meng-qi Yang, Xin Zhang, Dan Li, Yun-chang Liu, Zhi-yun Liao, Jiang-dong Sui, Ying Wang

**Affiliations:** Radiation Oncology Center, Chongqing University Cancer Hospital, College of Medicine, Chongqing University, Chongqing, China

**Keywords:** hnRNPA1, Oral squamous cell carcinoma, MARF1, Radioresistance, Alternative splicing

## Abstract

**Background:**

Radioresistance remains a major obstacle in oral squamous cell carcinoma (OSCC) therapy. This study aimed to elucidate the role of abnormal alternative splicing (AS) and key splicing factors in this process, focusing on their mechanistic contributions to DNA repair.

**Methods:**

We integrated RNA-sequencing data from irradiated OSCC mouse models with public datasets to profile splicing factor dynamics. Clinical relevance of hnRNPA1 was validated in an OSCC cohort. Functional roles were assessed via clonogenic survival, CCK-8, and xenograft assays. Mechanisms were delineated using co-immunoprecipitation, immunofluorescence, and analysis of MARF1 splicing and downstream DNA repair pathways.

**Results:**

hnRNPA1 was significantly upregulated in OSCC tissues and correlated with poor radiotherapy response. Its knockdown suppressed OSCC cell proliferation and impaired DNA double-strand break repair. Mechanistically, hnRNPA1 interacted with SF3B3 to inhibit exon 8 skipping of MARF1, promoting the oncogenic MARF1-L isoform. MARF1-L enhanced radioresistance by degrading PPP1R10, a negative regulator of Chk1, thereby activating homologous recombination repair (HR).

**Conclusions:**

These findings identified hnRNPA1 as a pivotal orchestrator of OSCC radioresistance, which, through an SF3B3-dependent MARF1 splicing switch and subsequent PPP1R10 degradation, activates HR repair. Targeting the hnRNPA1-SF3B3-MARF1 axis presents a novel therapeutic strategy to overcome radioresistance in OSCC.

**Supplementary Information:**

The online version contains supplementary material available at 10.1186/s13046-026-03697-4.

## Background

Oral squamous cell carcinoma (OSCC) ranks among the most prevalent and aggressive malignancies of the head and neck, characterized by high rates of local recurrence and mortality [1, 2]. Radiotherapy (RT) serves as a cornerstone in its therapeutic management, administered either as a definitive treatment or in combination with surgery and/or chemotherapy [3–5]. However, the development of radioresistance in a substantial proportion of patients severely compromises treatment efficacy, often leading to disease relapse and metastatic progression [6–8]. Therefore, uncovering the molecular drivers of radioresistance is imperative for improving clinical outcomes in OSCC.

Alternative splicing (AS), a crucial post-transcriptional mechanism, greatly expands proteomic diversity from a limited set of genes, with over 95% of human multi-exon genes undergoing this process [9–12]. Notably, aberrant AS patterns are a hallmark of cancer, occurring more frequently in tumors than in normal tissues [13–15]. Dysregulation of splicing factors is a key mechanism underlying these pathological AS events, which can reprogram cellular pathways to foster tumorigenesis, progression, and therapy resistance. For example, PTBP1 and SRSF1 have been shown to drive radioresistance in prostate and lung cancers, respectively [14, 16]. Despite these advances, the specific contributions of aberrant AS and its regulatory machinery to radioresistance in OSCC remain largely unexplored.

In this study, we aimed to identify key splicing factors involved in OSCC radioresistance. Through an integrated analysis of RNA-seq data from OSCC mouse models and public datasets, we identified heterogeneous nuclear ribonucleoprotein A1 (hnRNPA1) as significantly upregulated splicing factor in OSCC tissues. Although hnRNPA1, a core member of the hnRNP family, has previously been implicated in tumorigenesis—for example, through regulating PKM splicing to promote proliferation and metastasis in various cancers [17–19]—its role and mechanism in OSCC radioresistance are unknown. Here, we demonstrate that hnRNPA1 critically promotes radioresistance in OSCC. Mechanistically, hnRNPA1 cooperates with splicing factor 3B subunit 3 (SF3B3) to regulate the AS of meiosis regulator and mRNA stability factor 1 (MARF1), specifically by promoting the inclusion of exon 8. This splicing switch generates the MARF1-L isoform, which activates a downstream signaling cascade to enhance DNA damage repair. Our findings thus delineate a novel hnRNPA1-SF3B3-MARF1 axis that drives radioresistance, highlighting its potential as a therapeutic target for overcoming radioresistance in OSCC.

## Methods

### Cell culture and cell lines

HSC-3-M3 (purchased from Biospes Co., Ltd), CAL27 (obtained from the American Type Culture Collection (Manassas, VA, USA)), HSC-4 (purchased from Meisen CTCC), HSC-3 (obtained from the Japanese Collection of Research Bioresources Cell Bank(Osaka, Japan)), SCC25 (purchased from Biospes Co., Ltd), and MOC1 (purchased from Meisen CTCC) cell lines were cultured in DMEM (Gibco) supplemented with 10% fetal bovine serum (FBS, Gibco) and 1% penicillin/streptomycin (Gibco) at 37 °C in a humidified atmosphere containing 5% CO_2_. Regular testing via the EEZ-PCR mycoplasma test kit (BI) was conducted to ensure the absence of mycoplasma contamination. All cell lines were authenticated prior to use.

### Inhibitor treatments

OSCC cells were treated with the following pharmacological inhibitors: 25 nM of the PP1 inhibitor Okadaic acid (MCE, Cat. No. HY-N6785) for 24 h; 2 µM of the ATR inhibitor AZD6738 (MCE, Cat. No. AZD6738) for 24 h; and 1 µM of the CHK1 inhibitor MK8776 (MCE, Cat. No. MK8776) for 24 h.

### RNA extraction, qPCR, RNA-seq, and ONT Isoform quantitative full-length transcriptome sequencing

Total RNA was extracted using the RNA extraction kit (Takara). cDNA was synthesized from total RNA using a reverse transcription kit (Takara) following standard protocols. Quantitative real-time PCR (qPCR) was performed using a SYBR Green PCR kit (Takara) with specific primers, and GAPDH served as an internal control. Primers sequemces were listed in Table S9. For RNA-seq, total RNA was used for cDNA library construction and sequenced on an Illumina platform (Annoroad, Beijing, China). AS events were quantified using rMATS. Raw RNA-seq data were deposited in the Gene Expression Omnibus (GEO) database (GSE276977). For ONT Isoform quantitative full-length transcriptome sequencing, RNA integrity was jointly verified using Nanodrop, Qubit, agarose gel electrophoresis, and Agilent 2100. Nanodrop detection showed that the A260/A280 ratio of RNA samples was between 1.8 and 2.0, indicating extremely low protein contamination; the A260/A230 ratio was ≥ 2.0, suggesting no significant contamination by impurities such as salt ions and polysaccharides. Qubit detection results showed that the RNA concentration was ≥ 100 ng/µL and the total amount was ≥ 1 µg, meeting the minimum sample requirement for library construction. Agarose gel electrophoresis showed clear bands of 28 S rRNA and 18 S rRNA with no obvious degradation, and the band intensity ratio was close to 2:1, indicating good RNA integrity. Agilent 2100 detection showed that the RNA Integrity Number (RIN) was ≥ 8.0, further confirming that RNA was not degraded and could be used for subsequent full-length cDNA synthesis and library construction. Full-length cDNA libraries were constructed by reverse transcription with Oligo (dT) primers, followed by low-cycle PCR amplification and adapter ligation, and sequenced on FLO-PRO002 flow cells. Raw fast5 data were base-called using GUPPY v6.0.1 software to generate fastq files. Reads were normalized by RPM (Reads Per Million), with a coefficient of variation (CV) of transcript expression levels < 0.3 after RPM normalization, and visualized using in-house scripts.

### Clinical samples

Tumor and adjacent non-tumor tissues from 60 patients with OSCC were collected as tissue microarrays from Aifang Biotechnology Co., Ltd. (Hunan, China; Ethics approval no. HN20250401, with clinical information detailed in Table S2. Additionally, 29 biopsy samples were obtained from patients undergoing radical radiotherapy at the Affiliated Tumor Hospital of Chongqing University (Ethics approval no. CZLS2024017-A) with informed consent (Table S3). All samples were histologically confirmed by two experienced pathologists using hematoxylin and eosin (H&E) staining.

### Immunohistochemistry (IHC)

Histological Sect.  (4 μm) were deparaffinized and dehydrated after being baked overnight at 60 °C. Antigen retrieval and peroxidase blocking were performed before incubation with primary antibodies (Table S6) overnight at 4 °C, followed by secondary antibody incubation for 1 h at room temperature. Diaminobenzidine (DAB, ZSGB-BIO) was used for color development, and nuclei were counterstained with hematoxylin. Five fields of view were randomly selected at low magnification using a light microscope. Two blinded pathologists independently evaluated staining intensity and percentage of positive cells. The IHC score was calculated by multiplying the staining intensity (0–3, 0 = no staining; 1 = weak; 2 = moderate; 3 = strong) by the percentage score (0–4, 0 = < 1%; 1 = 1–25%; 2 = 26–50%; 3 = 51–75%; 4 = 76–100%). A median IHC score across all samples was used as the cutoff to stratify cases into high- and low-expression groups for hnRNPA1. For Ki-67 analysis, the percentage of positive nuclei was quantified in five randomly selected 200× magnification fields per tissue chip.

### Western blot (WB)

Cells were lysed in RIPA buffer on ice for 30 min and centrifuged at 12,000 × g for 20 min at 4 °C. Protein concentration was determined using a BCA kit (Beyotime). Equal amounts of protein were separated by SDS–PAGE, transferred onto PVDF membranes (Merck), blocked with 5% skim milk, and incubated overnight at 4 °C with primary antibodies (Table S6). After washing, membranes were incubated with HRP-conjugated secondary antibodies (Table S7) for 1 h at room temperature and visualized using ChemiDoc MP (Bio-Rad). Band intensities were quantified using ImageJ, with GAPDH as the loading control.

### Immunofluorescence (IF)

HSC-3 and SCC25 cells (1 × 10⁵ cells/mL) were seeded into 24-well plates, irradiated with 6 Gy X-rays, and fixed 12 h later with 4% paraformaldehyde for 30 min. Cells were permeabilized with 0.5% Triton X-100, blocked with 5% BSA, and incubated overnight at 4 °C with primary antibodies (Table S6). Fluorescent secondary antibodies (Table S7) were applied for 1 h in the dark, and nuclei were counterstained with DAPI (Solarbio). Images were acquired using a confocal laser scanning microscope (STELLARIS 5, Leica) and analyzed with ImageJ.

### HR / Non-Homologous End Joining (NHEJ) DR-GFP assay

HR and NHEJ activities were assessed using the pDR-GFP reporter system (Watertown, MA, Addgene plasmids #44026, #26477, #26475). HSC-3 and SCC25 cells were stably transfected with pDr-GFP. Upon 60% confluence, the stably transfected cells were transfected with the above plasmid. Restriction enzyme I-Sce1 cut the reporter plasmid and initiated GFP expression when the damage was repaired by HR and NHEJ. After transfection of plasmids for 48 h, GFP-positive cells were measured by flow cytometry using a Novocyte Advanteon (Agilent Biosciences) flow cytometer. The flow cytometry gating strategy was performed as follows: single intact cells were first gated on a dot plot using forward scatter (FSC) and side scatter (SSC) to exclude cellular debris, dead cells, and cell aggregates. Based on the single-cell gate, unstained cells or cells not transfected with the I-SceI plasmid served as a negative control to set the threshold for GFP fluorescence, thereby clearly distinguishing GFP-negative and GFP-positive cells. The percentage of GFP-positive cells within the single-cell population was quantified to reflect the efficiency of HR- or NHEJ-mediated DNA repair. Identical voltage and gating parameters were applied to all samples within the same cell line to ensure comparability between experimental groups. A total of three independent experiments were performed.

### Detection of cellular protein phosphatase 1(PP1)Activity

To measure cellular PP1 activity, HSC-3 and SCC25 cells from the control group, the MARF1-L overexpression group, and the MARF1-S overexpression group were collected. A PP1 Activity Colorimetric Assay Kit (Shanghai Halin, HL50313.1) was used for quantification. Briefly, total cellular protein was extracted using the lysis buffer provided in the kit and quantified. In the assay system, a PP2A-specific inhibitor was added to exclude interference from PP2A. Cell lysates containing equal amounts of protein were incubated with the specific substrate glycogen phosphorylase a at 30 °C for 10 min, allowing PP1 to catalyze the dephosphorylation reaction and release inorganic phosphate. The reaction was then terminated by adding stop solution, followed by the addition of malachite green color development solution to react with the free phosphate. After incubation at room temperature in the dark for 15 min, the absorbance was measured at 660 nm using a spectrophotometer. The absorbance values were converted to inorganic phosphate concentration using a synchronously generated phosphate standard curve. Sample activity was calculated according to the formula, and the final results are expressed as the amount of inorganic phosphate catalyzed per milligram of protein per minute (nmol Pi/mg protein/min). Three replicates were included for each sample.

### RNA interference and plasmid transfection

All small interfering RNAs (siRNAs) targeting genes and negative control (NC) siRNAs were synthesized by GenePharma Co.,Ltd (Shanghai, China). Transfections were performed using Lipofectamine RNAiMAX (Invitrogen) according to the manufacturer’s protocol. OSCC cells were seeded in six-well plates and incubated overnight to obtain 30% confluence before transfection. The transfection mixture was then incubated at room temperature for 15 min and added to each well, after which the cells were cultured in the appropriate medium for 24 h. The medium was subsequently replaced with fresh medium containing 10% FBS. Knockdown efficiency was verified by qRT–PCR and western blotting. Knockdown efficiency was verified by qRT–PCR and western blotting. Sequences were listed in Supplementary Table S8.

### Stable cell line construction

Lentiviral particles for hnRNPA1 knockdown or overexpression (sh-hnRNPA1, OE-hnRNPA1, sh-NC) were obtained from Hanbio (Shanghai, China), and MARF1 variants (MARF1-L, MARF1-S, sh-NC) from General Biol (Anhui, China). OSCC cells were transduced according to the manufacturer’s protocol and selected with puromycin. Stable lines were validated by qRT–PCR and western blotting. Sequences are provided in Table S8.

### X-ray radiation, CCK-8 and colony formation assay

HSC-3/SCC25 cells in the logarithmic growth phase were chosen and added to 96-well culture plates and exposed to varying doses of X-ray radiation (0, 2, 4, 6, 8 Gy). After treatment for 96 h, cell viability was assessed using the CCK-8 assay (Solarbio), and absorbance was measured at 450 nm (Biotek). Dose–response curves were generated using GraphPad Prism 8. For colony formation assay, cells were seeded in six-well plates, irradiated, and cultured for 14 days. Colonies were fixed with 4% paraformaldehyde, stained with 0.1% crystal violet, and counted using ImageJ. Each experiment was repeated at least three times independently.

### Co-IP, and Mass Spectrometry (MS)

Cell lysates were incubated with specific antibodies and protein A/G magnetic beads (MCE Bio). Immune complexes were washed, eluted, and analyzed by western blot or subjected to MALDI-TOF MS (Bruker Daltonics) for proteomic screening.

### RNA immunoprecipitation (RIP)

RIP assays were performed using the Magna RIP RNA-Binding Protein Immunoprecipitation Kit (Bersin Bio). Cell lysates were incubated overnight at 4 °C with antibody-conjugated or IgG control beads. Co-precipitated RNA was purified using the RNeasy MinElute Cleanup Kit (Qiagen) and analyzed by qPCR.

### RNA pull-down assays

Biotin-labeled RNA probes (Tsingke Bio, Beijing) were incubated with streptavidin magnetic beads, followed by OSCC cell lysates. After washing, bound proteins were eluted and analyzed by western blot. Probe sequences are shown in Table S8.

### Alkaline comet assay

DNA damage was evaluated using the comet assay kit (Beyotime) following IR (6 Gy). Cells were embedded in low-melting agarose, lysed, and subjected to alkaline electrophoresis (pH > 13, 18 V/cm). Comet tails were visualized with an Olympus IX51 microscope, and Olive tail moments were quantified using ImageJ. Experiments were repeated three times independently.

### Tumor xenograft models

All animal experiments were performed in accordance with the Guide for the Care and Use of Laboratory Animals and approved by the Animal Care and Use Committee of Chongqing University Cancer Hospital (approval no. CQCH-LAE-A0000202024). Female BALB/c nude mice aged 8–10 weeks were purchased from Jiangsu GemPharmatech and housed under specific pathogen-free (SPF) conditions. HSC-3 cells (1 × 10⁶) transduced with negative control or sh-hnRNPA1 lentivirus were mixed with Matrigel and injected subcutaneously into the right flank of mice. Tumor volumes were measured every 3 days and calculated using the formula: 0.5 × length × width². When tumors reached approximately 100 mm³ (around 10 days after implantation), mice were randomly assigned to non-irradiated or irradiated groups. The irradiation protocol consisted of daily fractions of 8 Gy for 3 consecutive days, with a total dose of 24 Gy. Mice were randomly divided into groups of six animals per group. A single-blind design was applied throughout the experiment: radiotherapy administration and subsequent indicator detection were performed by different investigators, and the analysts were blinded to group allocation during data collection and analysis to minimize subjective bias. Mice were sacrificed when tumor volumes reached approximately 1000 mm³.

### Statistical analysis

Statistical analyzes were performed using GraphPad Prism 8. Data were presented as mean ± SD. Differences between two groups were analyzed by unpaired Student’s t-test, and multiple comparisons were assessed using one-way ANOVA with Bonferroni correction. A p value < 0.05 was considered statistically significant (* *p* < 0.05; ** *p* < 0.01; *** *p* < 0.001; **** *p* < 0.0001). Each experiment was repeated at least three times independently.

## Results

### hnRNPA1 overexpression post-radiation correlates with poor prognosis and radiotherapy efficacy in OSCC

**Fig. 1:**
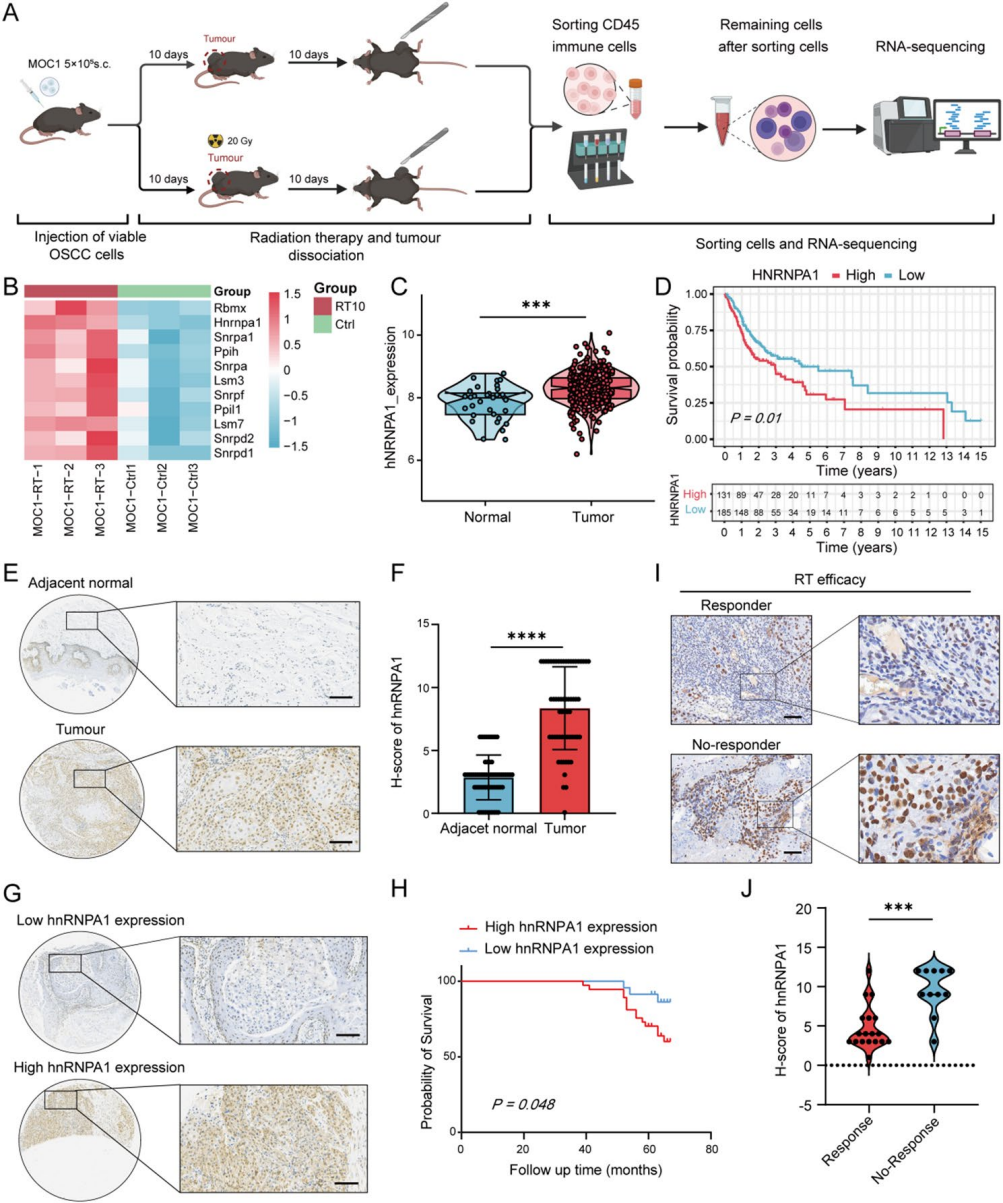
hnRNPA1 was upregulated in OSCC and associated with poor prognosis and reduced radiotherapy efficacy. **A** Workflow of RNA sequencing comparing tumors from control and post-radiotherapy OSCC mouse models, S.C., subcutaneous; (**B**) Heatmap showing eleven alternatively spliced molecules significantly elevated at the RNA level in post-radiotherapy OSCC tissues; (**C**) Expression levels of hnRNPA1 in tumor versus adjacent tissues from the TCGA-OSCC dataset; (**D**) Kaplan–Meier overall survival analysis stratified by high versus low hnRNPA1 expression in the TCGA-OSCC dataset; (**E**-**F**) Representative immunohistochemical staining and scoring analysis of hnRNPA1 expression in OSCC tissue microarrays, comparing tumor and adjacent tissues; (**G**-**H**) Overall survival curve analysis of OSCC tissue microarrays stratified by hnRNPA1 expression levels; (**I**) Immunohistochemical detection and (**J**) immunohistochemical scoring analysis of hnRNPA1 expression in OSCC tissues from patients undergoing radical radiotherapy at our institution; Data are presented as mean ± SD. Statistical significance was determined using two-tailed Student’s t-test or two-way ANOVA followed by Tukey’s post-hoc test, as appropriate. * *p* < 0.05; ** *p* < 0.01; *** *p* < 0.001; **** *p* < 0.0001; ns, not significant

To identify key determinants of radiotherapy response in OSCC, we established a subcutaneous tumor mouse model using MOC1 mouse OSCC cells. Tumors from control and irradiated groups were then harvested for RNA sequencing analysis, as outlined in Fig. 1A. By screening the expression profiles of 134 splicing factors (Table S1), we identified 11 factors that were significantly dysregulated at the mRNA level (screening criterion: log2FC > 0.5) following radiotherapy compared to non-irradiated tumors (Fig. 1B). Among these, hnRNPA1 was selected for further investigation due to its prominent upregulation and its previously undefined role in OSCC radioresistance.

Subsequent analysis of The Cancer Genome Atlas (TCGA)-OSCC dataset revealed significantly elevated hnRNPA1 expression in OSCC tissues compared with normal oral tissues (Fig. 1C). This finding was further validated using OSCC tissue microarrays (Table S2), which demonstrated marked overexpression of hnRNPA1 in tumor tissues relative to matched adjacent tissues (Fig. 1E–F). Critically, Kaplan-Meier survival analysis based on both TCGA dataset and our tissue microarrays consistently revealed that high hnRNPA1 expression was associated with significantly shorter overall survival in OSCC patients (Fig. 1D and H).

To directly evaluate the clinical relevance of hnRNPA1 in radiotherapy response, we performed a multivariate regression analysis on tumor specimens from patients with OSCC who underwent radical radiotherapy at our institution. Table S3 summarizes the baseline characteristics of these patients. Immunohistochemical (IHC) scoring of hnRNPA1 was significantly associated with poorer radiotherapy efficacy (Figs. 1I–J). Multivariate logistic regression analysis of the 29 patients revealed that high hnRNPA1 expression was closely correlated with non-response to radiotherapy [hazard ratio = 0.04 (95% confidence interval (CI): 0.01, 0.32), *P* = 0.002] (Table S4). Collectively, these results demonstrated that hnRNPA1 is aberrantly upregulated in OSCC and that its higher expression robustly predicted poorer radiotherapy sensitivity and unfavorable patients’ prognosis.

#### hnRNPA1 enhances the radioresistance of OSCC cells in vitro

Given the established role of hnRNPA1 in promoting tumor growth in various solid malignancies [20], we first sought to examine its function in OSCC. Western blot analysis across a panel of OSCC cell lines revealed varying protein levels of hnRNPA1, with HSC-3 cells exhibiting the highest and SCC25 cells showing the lowest expression, so these two cell lines were consequently selected for subsequent experiments (Figure S1A). We next investigated whether RT itself influenced hnRNPA1 expression. Notably, both mRNA and protein levels of hnRNPA1 were significantly upregulated following IR in a dose-dependent manner (2, 4, 6, and 8 Gy) compared with untreated controls (Figure S1B–C).

**Fig. 2:**
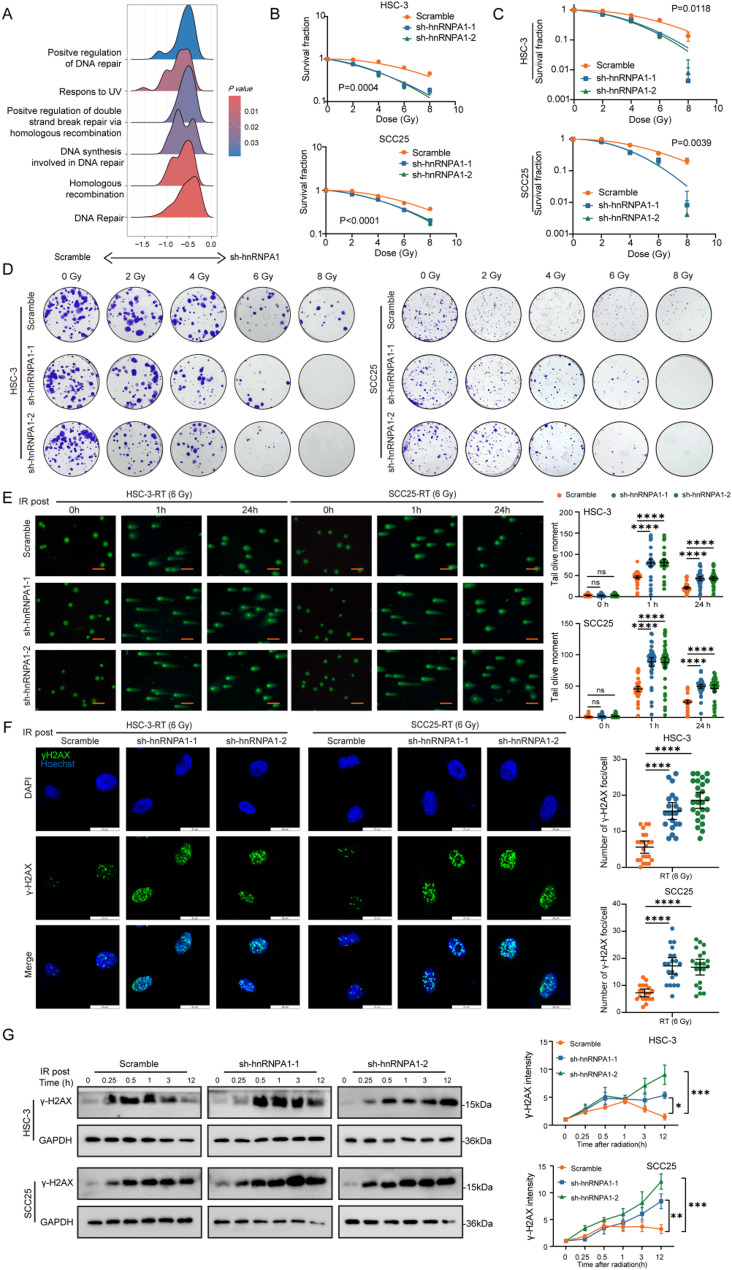
Knockdown of hnRNPA1 enhanced radiosensitivity in OSCC cells in vitro. **A** GSEA enrichment of differentially expressed genes between sh-hnRNPA1 and control cells; (**B**) CCK-8 assay assessing survival rate of OSCC cells exposed to increasing radiation doses (0, 2, 4, 6, 8 Gy) for 96 h; (**C**-**D**) Clonogenic assays evaluating colony-forming ability of OSCC cells following 2 weeks of IR at indicated doses; (**E**) Comet assays detecting DNA damage at 1 and 24 h post-6 Gy IR (scale bar, 20 μm); (**F**) Immunofluorescence assays of γ-H2AX foci at 12 h post-6 Gy IR (scale bar, 20 μm); (**G**) Western blot analysis of γ-H2AX protein levelsat indicated time points (0.25–12 h) after 6 Gy IR. Statistical significance was assessed by two-tailed Student’s t-test or two-way ANOVA with Tukey’s post hoc test. * *p* < 0.05; ** *p* < 0.01; *** *p* < 0.001; **** *p* < 0.0001; ns, not significant

To further explore its functional role, HSC-3 and SCC25 cells were transduced with lentiviruses carrying shRNA or OE constructs targeting hnRNPA1. Knockdown and overexpression efficiency were validated by qPCR and Western blotting (Figure S1D–G). RNA sequencing followed by gene set enrichment analysis (GSEA) performed on control versus hnRNPA1-knockdown cells revealed a significant enrichment of DNA damage repair and homologous recombination pathways in the control group (Fig. 2A), suggesting that hnRNPA1 deficiency impairs DNA repair signaling. To investigate the potential involvement of hnRNPA1 in the response to IR, CCK-8 assay and clone formation assay demonstrated that hnRNPA1 depletion significantly reduced OSCC cell proliferation and colony formation following IR, whereas its overexpression conferred the opposite effect (Fig. 2B-D and Figure S2A-B).

We further directly assessed the impact of hnRNPA1 on IR-induced genomic instability, Comet assays conducted at 1 and 24 h post-6 Gy IR showed that hnRNPA1-knockdown cells sustained significantly more DNA damage, as evidenced by longer comet tails, compared to controls. Conversely, hnRNPA1-overexpressing cells exhibited less damage (Fig. 2E, Figure S2C). Consistent with these findings, IF analysis demonstrated a marked increase in γ-H2AX foci—a key marker of DNA double-strand breaks—in hnRNPA1-depleted cells 12 h after IR, while overexpressing cells showed a reduction (Fig. 2F, Figure S2D-E). This trend was corroborated by Western blot analysis, which confirmed elevated and sustained γ-H2AX protein levels in knockdown cells and reduced levels in overexpressing cells across multiple time points following IR (Fig. 2G, Figure S2F-G). Together, these results indicated that hnRNPA1 promoted OSCC cell proliferation and enhanced resistance to radiotherapy in vitro by facilitating DNA damage repair.

### hnRNPA1 enhances radiotherapy tolerance of OSCC cells in vivo

**Fig. 3:**
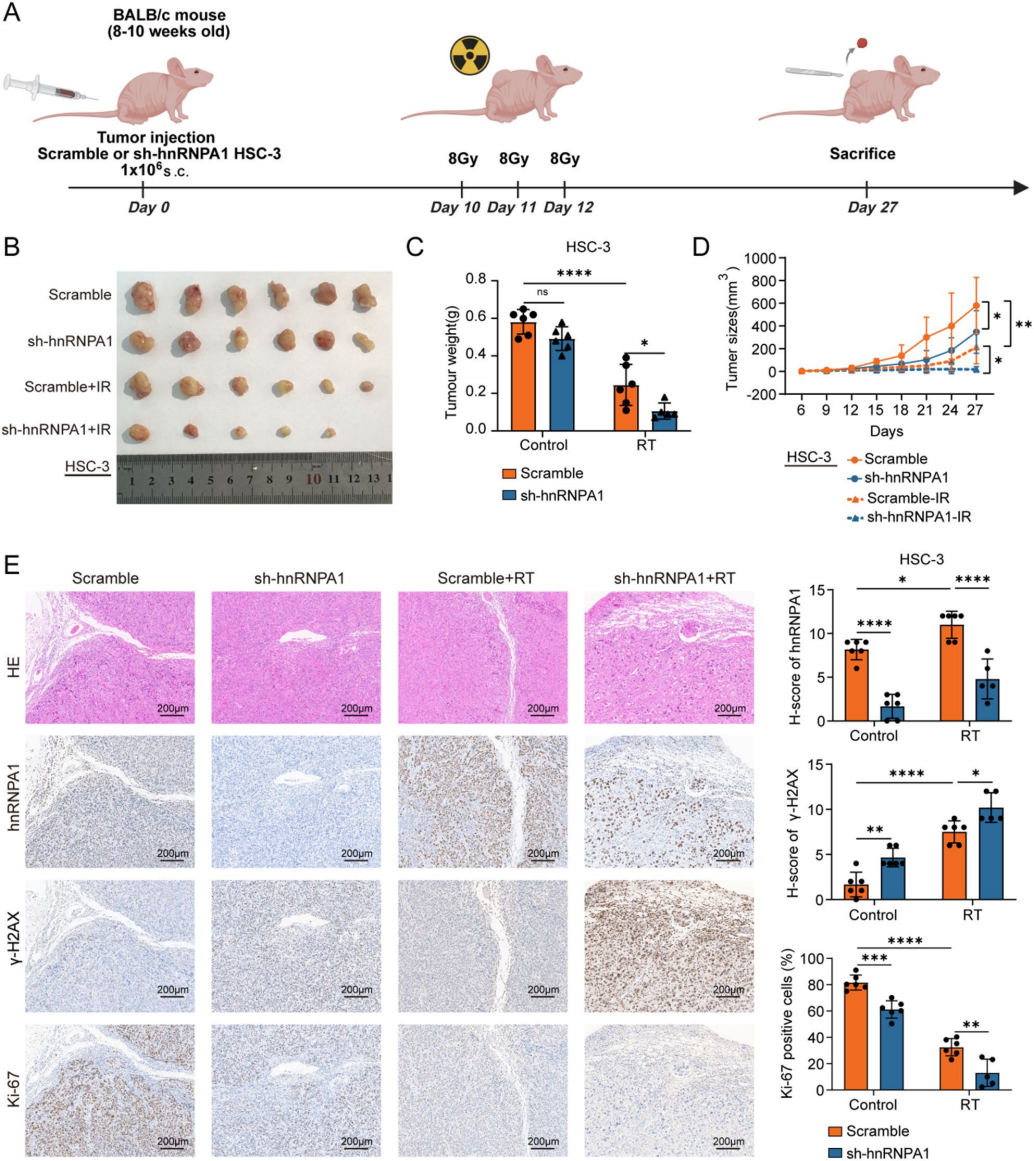
hnRPNA1 contributed to OSCC radioresistance in vivo. **A** Schematic of the xenograft mouse experimental workflow, S.C.,subcutaneous; (**B**) Images of tumors from Scramble and sh-hnRNPA1 groups; (**C**) Quantification of tumor weights; (**D**) Tumor growth curves over time; (**E**) H&E staining and immunohistochemistry for Ki-67, hnRNPA1, and γ-H2AX, with corresponding IHC scoring.Scale bar, 200 μm. Data are presented as mean ± SD. Statistical significance was determined by two-tailed Student’s t-test or two-way ANOVA with Tukey’s post hoc test. * *p* < 0.05; ** *p* < 0.01; *** *p* < 0.001; **** *p* < 0.0001; ns, not significant

To validate our in vitro findings, we established a xenograft mouse model by subcutaneously implanting BALB/c-nu nude mice with HSC-3 cells stably transfected with sh-hnRNPA1 or control lentivirus. When tumors became palpable 10 days after implantation, the mice were subjected to local tumor irradiation at a fractionated dose of 8 Gy daily for 3 consecutive days (Fig. 3A). Tumor growth was markedly suppressed following ionizing radiation treatment. Compared with the control group, hnRNPA1 knockdown further inhibited tumor growth, resulting in more significant reductions in xenograft tumor volume and weight after irradiation (Figs. 3B–D).

Consistent with our in vitro results, immunohistochemical analysis of xenograft tissues revealed significantly lower Ki-67 expression in the sh-hnRNPA1 group than in the control group. In addition, the expression of γ-H2AX was higher in the sh-hnRNPA1 group than in the control group following ionizing radiation (Fig. 3E). Collectively, these results demonstrate that hnRNPA1 serves as a critical regulator of radioresistance in OSCC.

### Knockdown of hnRNPA1 induces global alterations in AS events

To elucidate the molecular mechanism by which hnRNPA1 promotes radiation resistance, we performed Oxford Nanopore Technologies (ONT)-based full-length transcriptome sequencing to analyze AS events in OSCC cells with or without hnRNPA1 knockdown. A total of 604 differential AS events were identified and classified into seven types, with skipped exons (SE) being the most predominant, indicating that hnRNPA1 mainly regulates exon skipping (Figure S3A). Further analysis revealed that hnRNPA1 functioned as both a splicing activator and repressor, mediating comparable proportions of exon inclusion and exclusion events (Figure S3B). To validate the reliability of the sequencing data, we selected several top-ranking hnRNPA1-dependent AS events for confirmation by qPCR, which consistently supported the ONT findings (Figure S3C). These results firmly establish hnRNPA1 as a bidirectional regulator of splicing in OSCC cells. We further analyzed We next conducted Gene Ontology (GO) analysis to explore the biological functions of genes whose splicing was altered by hnRNPA1 knockdown. Notably, these hnRNPA1-regulated AS targets were significantly enriched in pathways related to DNA damage response and double-strand break repair (Figure S3D). This finding provides mechanistic support for our hypothesis that hnRNPA1 promotes radioresistance in OSCC by reprogramming the AS landscape of genes critical for DNA repair processes.

### hnRNPA1 facilitates MARF1 exon 8 inclusion through direct pre-mRNA binding

**Fig. 4:**
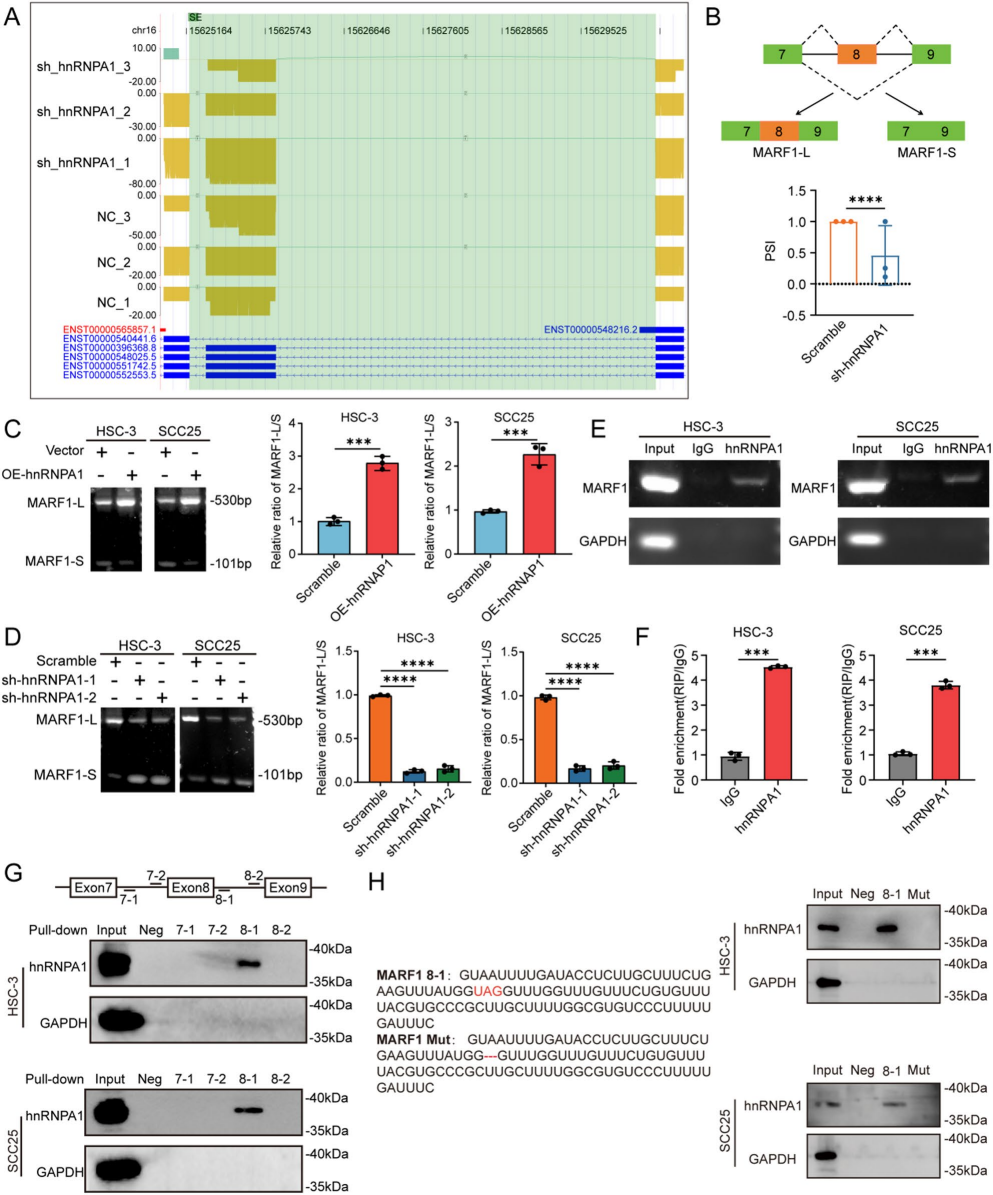
hnRNPA1 facilitated MARF1 exon 8 inclusion via direct binding to pre-mRNAs. **A** Distribution of reads containing splicing information: Distribution of reads supporting the MARF1 exon-skipping event, generated from ONT sequencing data. Compared with the conventional MARF1 read distribution, two additional graphical elements are included: green boxes denote the genomic region harboring the splicing event, and connecting lines with numbers indicate the counts of sequencing reads supporting the corresponding splicing site; (**B**) Statistical analysis of the inclusion level of exon 8 in MARF1 (represented by PSI values) between the sh-hnRNPA1 and control groups, based on ONT sequencing data; (**C**-**D**) Representative agarose gel electrophoresis and quantification of MARF1 exon 8 inclusion in OSCC cells overexpressing or silencing hnRNPA1, respectively, shown as MARF1-L/MARF1-S ratios; (**E**-**F**) RIP analysis showing enrichment of MARF1 pre-mRNA in hnRNPA1-immunoprecipitated samples compared with controls; (**G**-**H**) RNA pull-down followed by western blot demonstrating specific interaction between hnRNPA1 and intronic fragment 8 − 1 of MARF1 pre-mRNA. Statistical significance was determined by two-tailed Student’s t-test or two-way ANOVA with Tukey’s post hoc test. * p < 0.05; ** *p* < 0.01; *** *p* < 0.001; **** *p* < 0.0001; ns, not significant

To investigate how hnRNPA1 modulates DNA repair through alternative splicing, we focused on differentially spliced genes enriched in the double-strand break repair pathway. Among these candidates, MARF1 was prioritized for in-depth analysis based on its exon-skipping splicing pattern and the most significant change in Percent Spliced In (PSI) between the sh-hnRNPA1 and control groups (Table S5). Interrogation of our ONT sequencing data unequivocally demonstrated that hnRNPA1 promotes inclusion of exon 8 in MARF1 transcripts (Fig. 4A-B). This regulatory effect was functionally validated using orthogonal approaches. Consistent with the sequencing data, qPCR analysis coupled with agarose gel electrophoresis in OSCC cells demonstrated that hnRNPA1 overexpression increased exon 8 inclusion, whereas hnRNPA1 knockdown favored exon skipping (Fig. 4C-D). To determine whether hnRNPA1 regulated MARF1 splicing via direct RNA binding, we conducted RIP assays, which showed significant enrichment of MARF1 pre-mRNA in hnRNPA1 immunoprecipitates compared with controls (Fig. 4E-F). Meanwhile, the canonical hnRNPA1-binding core motif UAGGGA/U (often simplified as UAGGG, UAGG, or UAG) is widely distributed in the intronic regions upstream and downstream of exon 8 in MARF1 pre-mRNA [21–23]. To further pinpoint the functional binding regions of hnRNPA1 on MARF1 pre-mRNA, we designed and synthesized four independent oligonucleotide fragments derived from intronic sequences flanking exon 8. RNA pull-down assays revealed that hnRNPA1 directly binds to the 8 − 1 region (Fig. 4G), whereas deletion mutation of the UAG motif within the 8 − 1 region markedly impaired its association with hnRNPA1 (Fig. 4H). These findings collectively demonstrated that hnRNPA1 regulated exon 8 inclusion in MARF1 by directly binding to a specific motif in its pre-mRNA.

#### hnRNPA1 interacts with SF3B3 to regulate MARF1 splicing in OSCC

**Fig. 5:**
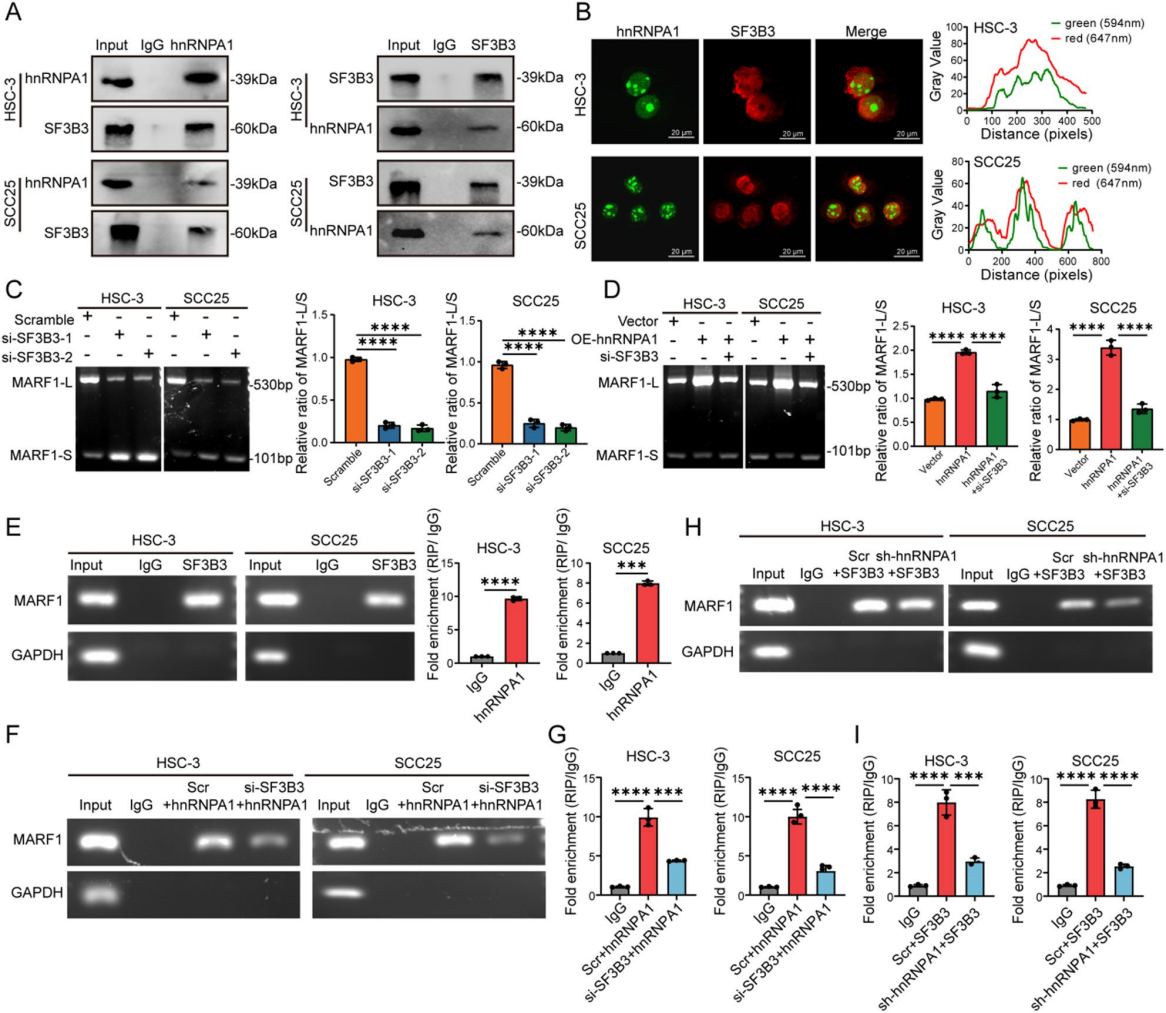
hnRNPA1-SF3B3 Interaction Modulated MARF1 Splicing in OSCC. **A **Co-immunoprecipitation (Co-IP) analysis demonstrating the interaction between hnRNPA1 and SF3B3in OSCC cells using anti-hnRNPA1 antibody and an anti-SF3B3 antibody; (**B**) Immunofluorescence demonstrating the subcellular co-localization of hnRNPA1 (green) and SF3B3 (red) in OSCC cells. Scale bar, 20 μm; (**C**) Representative gel electrophoresis image and quantitative analysis of the MARF1-L/MARF1-S ratio in SF3B3 knockdown OSCC cells determined by qPCR; (**D**) Representative gel electrophoresis image and quantitative analysis of the MARF1-L/MARF1-S ratio in hnRNPA1-overexpressing (OE-hnRNPA1) OSCC cells and hnRNPA1-overexpressing cells with SF3B3 knockdown (OE-hnRNPA1 + si-SF3B3) determined by qPCR; (**E**) Representative images and quantitative analysis of MARF1 pre-mRNA enrichment in SF3B3 immunoprecipitates from RIP assay in OSCC cells using anti-SF3B3 antibody; (**F**) Representative images of MARF1 pre-mRNA enrichment in hnRNPA1 immunoprecipitates from RIP assay in scramble and SF3B3-knockdown OSCC cells using anti-hnRNPA1 antibody; (**G**) Quantitative analysis of MARF1 pre-mRNA enrichment in hnRNPA1 immunoprecipitates from RIP assays in scramble control and SF3B3-knockdown OSCC cells using anti-hnRNPA1 antibody; (**H**) Representative images of MARF1 pre-mRNA enrichment in SF3B3 immunoprecipitates from RIP assay in scramble and hnRNPA1-knockdown OSCC cells using anti-SF3B3 antibody; (**I**) Quantitative analysis of MARF1 pre-mRNA enrichment in SF3B3 immunoprecipitates from RIP assay in scramble control and hnRNPA1-knockdown OSCC cells. * *p* < 0.05; ** *p* < 0.01; *** *p* < 0.001; **** *p* < 0.0001; ns, not significant

Since hnRNPA1 is known to function as a splicing regulator rather than a core spliceosomal component [24, 25], we sought to identify its functional partners that mediate alternative splicing in OSCC. Using immunoprecipitation coupled with MS in HSC-3 cells, we identified 826 proteins that interacted with hnRNPA1. GO enrichment analysis revealed strong enrichment for RNA splicing-related proteins, reinforcing hnRNPA1’s role in splicing regulation (Figure S4A). Among the 58 of proteins associated with AS (Figure S4B), members of the SNRNP family exhibited strong interactions, with SF3B3 showing the highest sequence coverage and thus selected for further investigation (Figure S4C). Co-IP confirmed a specific interaction between hnRNPA1 and SF3B3 (Fig. 5A), and IF demonstrated their co-localization within the nucleus of OSCC cells (Fig. 5B). Functional studies indicated that knockdown of SF3B3 significantly enhanced the radiosensitivity of OSCC cells, as shown by reduced clonogenic survival and increased γ-H2AX levels upon irradiation (Figures S4D-G). We next examined whether SF3B3 is involved in hnRNPA1-mediated MARF1 splicing. Depletion of SF3B3 promoted skipping of exon 8 in MARF1 pre-mRNA, phenocopying the effect of hnRNPA1 knockdown (Fig. 5C). RIP assays using anti-SF3B3 antibody further revealed significant enrichment of MARF1 pre-mRNA in SF3B3 immunoprecipitates, suggesting that SF3B3 directly participates in MARF1 splicing regulation (Fig. 5E). To determine whether hnRNPA1 depends on SF3B3 to regulate MARF1 splicing, we overexpressed hnRNPA1 in SF3B3-deficient cells. Both qPCR and gel electrophoresis showed that SF3B3 knockdown abolished hnRNPA1-induced exon 8 inclusion (Fig. 5D). Consistently, RIP assays using anti-hnRNPA1 antibody in SF3B3-decicient cells showed diminished enrichment of MARF1 pre-mRNA (Fig. 5F-G). Reciprocally, RIP assays using anti-SF3B3 antibody in hnRNPA1-depleted cells also revealed impaired SF3B3 binding to MARF1 pre-mRNA (Fig. 5H-I). These results suggested that hnRNPA1 recruits SF3B3 to MARF1 pre-mRNA, and this cooperative interaction is essential for regulating exon 8 inclusion, revealing a functionally coupled mechanism underlying hnRNPA1-mediated splicing regulation in OSCC.

### The radioresistance function of hnRNPA1 depends on MARF1-L expression

**Fig. 6:**
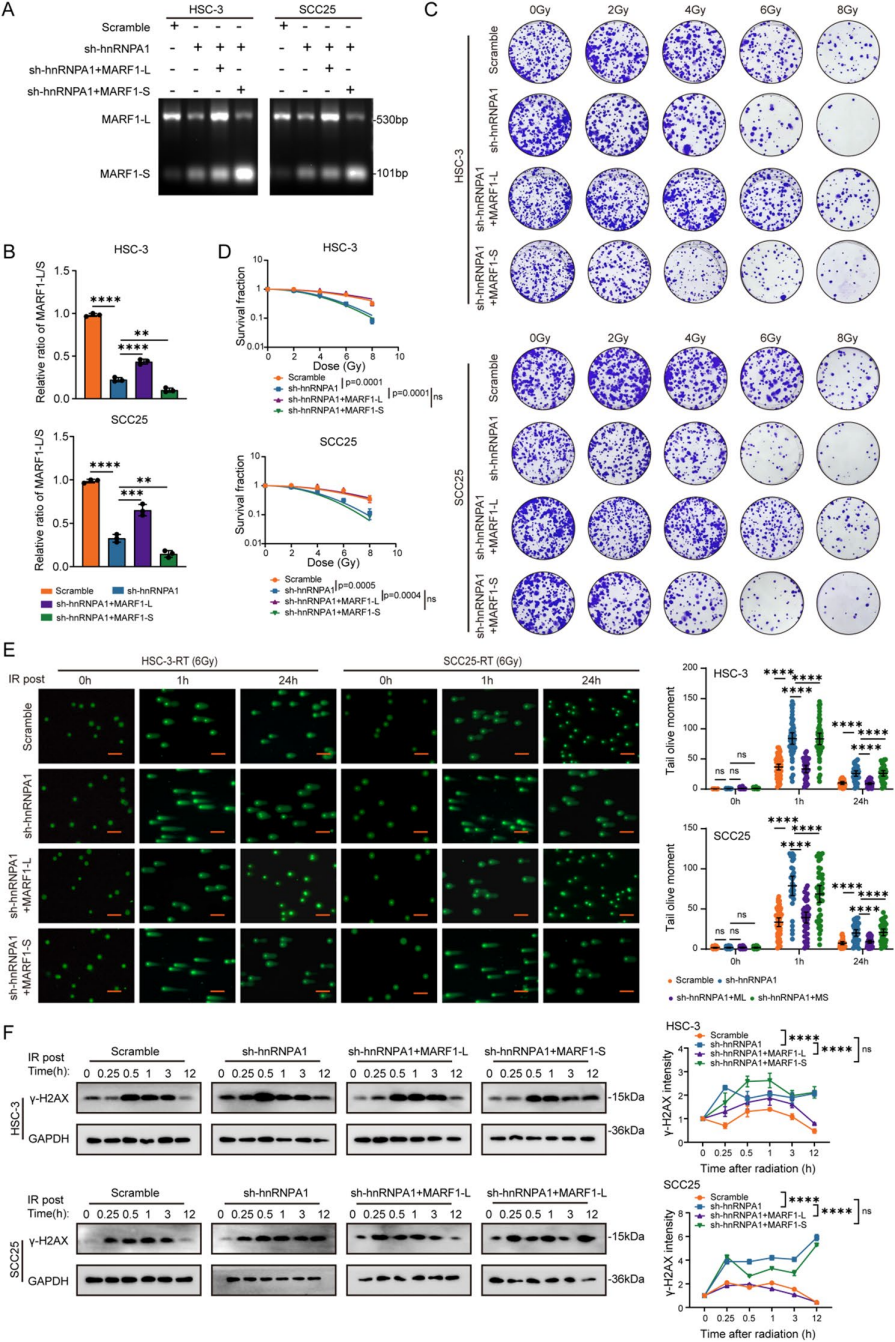
hnRNPA1-driven radioresistance in OSCC depended on MARF1-L expression. **A** Representative gel electrophoresis image and (**B**) qPCR analysis showing MARF1-L/MARF1-S ratios in OSCC cells from the scramble, sh-hnRNPA1, sh-hnRNPA1 + MARF1-L, and sh-hnRNPA1 + MARF1-S groups; (**C**) Clonogenic survival assays and (**D**) quantification of OSCC cells from the indicated groups following IR at 0, 2, 4, 6, and 8 Gy; (**E**) Comet assays detecting DNA damage in OSCC cells from the indicated groups at 1 and 24 h after 6 Gy IR (scale bar, 20 μm); (**F**) Western blot analysis of γ-H2AX expression in OSCC cells from the indicated groups at 0.25, 0.5, 1, 3, and 12 h after 6 Gy IR. Statistical significance was determined by two-tailed Student’s t-test or two-way ANOVA followed by Tukey’s post hoc test, as appropriate. * *p* < 0.05; ** *p* < 0.01; *** *p* < 0.001; **** *p* < 0.0001; ns, not significant

To delineate the functional contributions of the two MARF1 isoforms to radioresistance, we independently overexpressed MARF1-L and MARF1-S in OSCC cells, with successful isoform-specific expression confirmed by qPCR (Figure S5A). Functional assessment revealed a striking divergence: MARF1-L markedly enhanced the clonogenic capacity of OSCC cells following IR, whereas MARF1-S exhibited no such protective effect (Figure S5B). This isoform-specific function was further corroborated at the DNA damage level; Western blot analysis demonstrated that MARF1-L, but not MARF1-S, reduced γ-H2AX expression in irradiated cells (Figure S5C), indicating a specific role for MARF1-L in mitigating radiation-induced DNA damage. We next investigated whether MARF1-L operates within the hnRNPA1 pathway to promote radioresistance. In hnRNPA1-knockdown OSCC cells, we reconstituted expression of either MARF1-L or MARF1-S (Fig. 6A-B). Colony formation assays showed that MARF1-L, but not MARF1-S, partially rescued the radiosensitivity phenotype induced by hnRNPA1 depletion (Fig. 6C-D). Comet assays provided direct evidence for DNA repair, showing that MARF1-L expression in hnRNPA1-deficient cells resulted in significantly shorter comet tails at both 1 and 24 h post-irradiation, indicating accelerated DNA damage repair. In contrast, MARF1-S expression had no effect on DNA damage levels (Fig. 6E). In line with these findings, Western blot analysis showed that MARF1-L mitigated the accumulation of γ-H2AX triggered by hnRNPA1 knockdown after IR (Fig. 6F). In summary, these findings indicated that hnRNPA1 promoted the radioresistance of OSCC cells primarily through regulating the production of the MARF1-L isoform.

### hnRNPA1 promotes DNA repair via MARF1-L–mediated degradation of PPP1R10 mRNA to enhance Chk1 phosphorylation

**Fig. 7:**
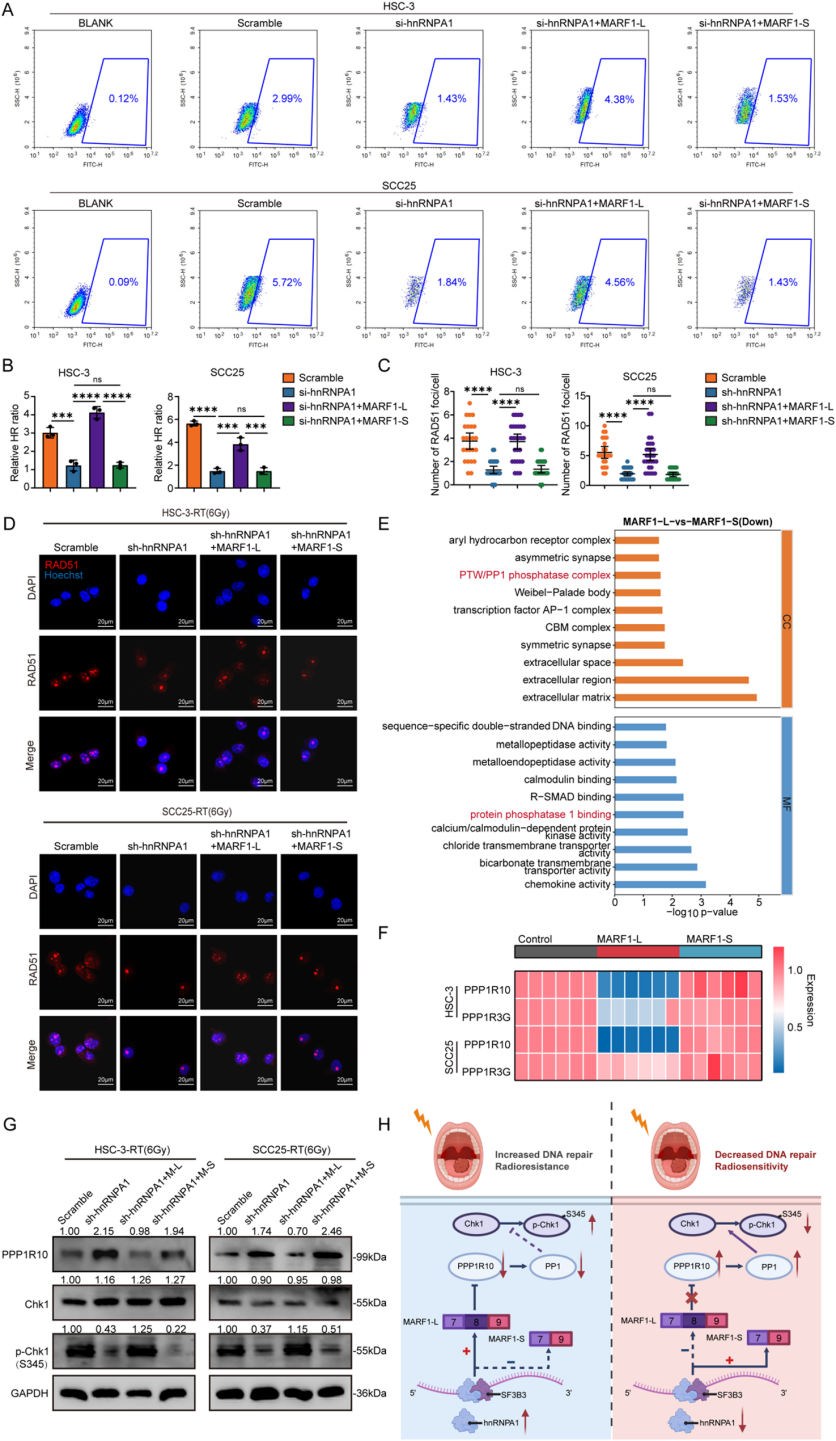
hnRNPA1 promoted DNA repair through MARF1-L–mediated PPP1R10 mRNA degradation and activation of Chk1 phosphorylation. **A**-**B** Homologous recombination (HR) repair efficiency in OSCC cells transduced with scramble, sh-hnRNPA1, sh-hnRNPA1 + MARF1-L or sh-hnRNPA1 + MARF1-S constructs; (**C**) Representative immunofluorescence images and (**D**)quantification assessing RAD51 foci formation in the indicated OSCC cell groups at 12 h after 6 Gy IR. Scale bar, 20 μm; (**E**) Enrichment analysis of Gene Ontology terms for downregulated differential genes (MARF1-L vs. MARF1-S groups), CC: Cellular Component, MF: Molecular Function. **F** Heatmap of qPCR analysis of PPP1R10 and PPP1R3G mRNA levels in control, MARF1-L, and MARF1-S OSCC cells. **G** Western blot analysis of PPP1R10, Chk1, p-Chk1 (S345) protein levels in the indicated OSCC cells at 12 h post-IR; (**H**) Schematic model illustrating how hnRNPA1 facilitated radioresistance by promoting MARF1-L–dependent degradation of PPP1R10 mRNA and subsequent activation of CHK1 signaling Statistical significance was determined by two-tailed Student’s t-test or two-way ANOVA followed by Tukey’s post hoc test, as appropriate. * *p* < 0.05; ** *p* < 0.01; *** *p* < 0.001; **** *p* < 0.0001; ns, not significant

MARF1 was an RNA-binding protein implicated in the regulation of meiosis, RNA stability, and RNA decay [26, 27]. Our results demonstrated that hnRNPA1 promoted radiation resistance in OSCC cells by regulating the MARF1-L transcript, yet the downstream mechanism remained unclear. To address this, we employed NHEJ and HR reporter (DR-GFP) assays in control and hnRNPA1-deficient OSCC cells. hnRNPA1 depletion markedly impaired HR efficiency, aligning with our prior transcriptomic data (Figure S6A and Fig. 2A). Importantly, reintroduction of MARF1-L, but not MARF1-S, partially restored HR activity in hnRNPA1-knockdown cells (Fig. 7A-B). Consistent with these results, IF analysis of RAD51 foci revealed that loss of hnRNPA1 reduced RAD51 assembly following IR, which was effectively rescued by MARF1-L overexpression, while MARF1-S had no effect (Fig. 7C-D). To further delineate the functional differences between the MARF1-L and MARF1-S, we conducted RNA sequencing in HSC-3 cells expressing each isoform. GO enrichment analysis of the downregulated genes in MARF1-L–expressing cells compared to MARF1-S–expressing cells revealed significant enrichment of terms related to protein phosphatase 1 (PP1) in both the Cellular Component (CC) and Molecular Function (MF) categories (Fig. 7E). Among these, PPP1R10 (protein phosphatase 1 regulatory subunit 10) and PPP1R3G (protein phosphatase 1 regulatory subunit 3G) emerged as key candidates. Subsequent qPCR validation in two independent OSCC cell lines confirmed that PPP1R10 expression was substantially reduced in MARF1-L–expressing cells relative to both control and MARF1-S groups (Fig. 7F).

Based on previous studies suggesting that PPP1R10 can modulate Chk1 phosphorylation by regulating PP1 activity [49], we first measured PP1 activity in OSCC HSC-3 and SCC25 cells using a PP1 activity colorimetric assay. The results showed that overexpression of MARF1-L significantly inhibited PP1 activity, while overexpression of MARF1-S had no such effect (Figure S7A). Furthermore, upon inhibition of PP1 activity with okadaic acid (25 nM, 24 h treatment), Western blot analysis revealed that the promotion of Chk1 phosphorylation at Ser345 by MARF1-L was abolished (Figure S7B-C). Co-immunoprecipitation experiments confirmed an interaction between PPP1R10 and CHK1(Figure S7D). Functionally, Western blot results demonstrated that knockdown of PPP1R10 enhanced Chk1 Ser345 phosphorylation, whereas overexpression of PPP1R10 reduced phosphorylation at this site (Figure S7E).

To systematically elucidate the relationship within the hnRNPA1–MARF1–PPP1R10–Chk1 axis, we examined Chk1 phosphorylation levels after irradiation by Western blot. Knockdown of hnRNPA1 significantly reduced radiation-induced Chk1 phosphorylation, whereas PPP1R10 knockdown enhanced it (Figure S7F). Further experiments revealed that hnRNPA1 deficiency upregulated PPP1R10 protein and promoted Chk1 phosphorylation, an effect that could be reversed by restoring MARF1-L expression (Fig. 7G and Figure S7G). Subsequently, using a DR-GFP-HR reporter assay combined with ATR/CHK1 inhibitor treatment (ATR inhibitor, 2 µM final concentration, 24 h; Chk1 inhibitor, 12 µM, 24 h), we found that hnRNPA1 knockdown reduced homologous recombination repair efficiency, while MARF1-L overexpression increased repair efficiency and rescued the repair defect caused by hnRNPA1 knockdown. Inhibitor treatment reduced repair efficiency to baseline levels in all groups without intergroup differences, indicating that the regulation of DNA repair by hnRNPA1 and MARF1-L depends on the ATR/CHK1 signaling pathway (Figure S7H).

In summary, this study reveals a novel regulatory axis that activates HR in OSCC: hnRNPA1 recruits SF3B3 to promote the generation of the MARF1-L isoform, which degrades PPP1R10, thereby releasing its inhibition of PP1, enhancing Chk1 phosphorylation, and promoting CHK1-mediated HR (Fig. 7H).

## Discussion

AS dysregulation is implicated in oncogenic networks, but its role in OSCC radioresistance lacked mechanistic insights. Our study demonstrates that hnRNPA1 serves as a critical driver of OSCC radioresistance by orchestrating a novel SF3B3-dependent MARF1 splicing switch, thereby advancing our understanding of AS mechanisms in cancer therapy resistance. We provide evidence that hnRNPA1, in concert with SF3B3, binds directly to MARF1 pre-mRNA to promote the inclusion of exon 8, generating the MARF1-L isoform that is essential for promoting HR. This hnRNPA1-MARF1-L axis represents a crucial mechanism that bridges dysregulated splicing with enhanced DNA damage repair capability, offering a fresh perspective on therapeutic resistance in OSCC.

RNA splicing was a tightly regulated and multifaceted process orchestrated by the spliceosome and its associated regulatory proteins [28–30]. As a member of the hnRNP family, hnRNPA1 primarily regulated AS by binding to specific RNA sequences and controlling exon inclusion or skipping, thereby influencing diverse aspects of tumor biology [31–33]. In this study, we provided the first evidence that hnRNPA1 overexpression promoted radioresistance in OSCC through regulation of MARF1 exon skipping, further extending its known functions in cancer progression. Comprehensive analysis of hnRNPA1-related studies suggested that hnRNPA1 may exhibit distinct biological roles and clinical relevance across different tumor types [20, 34–36]. One potential reason for these differences is the tumor-specific hnRNPA1-regulated AS networks. For example, our previous data demonstrated that phosphorylation of hnRNPA1 at Ser95 and Ser192 by DNA-PKcs during mitosis was required for its ability to bind single-stranded telomeric DNA and displaced RPA [37]. Additionally, hnRNPA1 interactd with SRSF2 to regulate AUF1 AS and thereby modulate cisplatin sensitivity in ovarian cancer [38]. Consistent with its dual regulatory capacity, our sequencing data revealed that hnRNPA1 knockdown in OSCC induced comparable levels of exon skipping and inclusion events, suggesting that hnRNPA1 could function as either an activator or repressor of AS in a transcript-specific manner.

DNA damage repair was a key mechanism driving tumor cell resistance to radiotherapy [39–41]. MARF1 is an evolutionarily conserved RNA-binding protein containing multiple RNA recognition motifs, including RRM and OST domains, as well as a nuclease-related domain [42]. MARF1 primarily regulated mRNA stability and translation, playing essential roles in germ cell development [27]. In mammals, such as mice, MARF1 maintained meiotic arrest in oocytes and ensured proper oocyte maturation by degrading or inhibiting the translation of specific mRNAs, particularly those involved in the cell cycle and meiotic progression [26, 43]. Maintaining genomic integrity in germ cells was critical for hereditary stability, requiring precise repair of meiotic or exogenous DNA lesions, including double-strand breaks and oxidative damage [44, 45]. Failure to resolve such lesions could result in chromosomal abnormalities may lead to embryonic lethality or genetic disorders [46]. MARF1 had been extensively linked to mRNA decay [27]and was annotated in the GO DNA repair pathway, suggesting a potential role in DNA repair, although direct evidence had been lacking. Our data revealed that hnRNPA1 significantly inhibited exon 8 skipping in MARF1 pre-mRNA, and this skipping event had a substantial impact on transcript abundance. Functional assays revealed that the full-length MARF1 transcript containing exon 8 significantly enhanced radioresistance in OSCC cells, while the truncated MARF1 isoform lacking exon 8 fails did not. This discrepancy may arise from the inactive mRNA decay capacity of MARF1-S, which could regulate key genes or pathways involved in DNA repair. Thus, our findings uncovered novel functional and regulatory mechanisms of MARF1 splice variants in OSCC.

Although splicing factors were critical regulators of pre-mRNA splicing, the sequences of their binding sites were highly conserved and could not fully account for AS events in cancer cells [47, 48]. Additional cofactors that mediate the interaction between splicing factors and pre-mRNA may contribute to cancer-specific AS [47, 49, 50]. For example, PTBP1 interacted with hnRNPA1 to modulate PKM AS, thereby promoting the proliferation, migration, and metastasis of colorectal cancer cells in vitro and in vivo [19]. Similarly, RALY and SF3B3 synergistically regulated the MTA1 splicing switch, reducing MTA1-S levels and alleviating its inhibitory effects on cholesterol synthesis genes, which enhanced HCC cell proliferation [51]. In this study, we found that hnRNPA1 regulated MARF1 AS in an SF3B3-dependent manner. Co-IP experiments demonstrated a physical interaction between hnRNPA1 and SF3B3. Both proteins associated with MARF1 pre-mRNA and promoted the splicing switch from MARF1-S to MARF1-L in OSCC cells. Notably, SF3B3 depletion markedly reduced hnRNPA1 binding to MARF1 pre-mRNA, and conversely, hnRNPA1 knockdown diminished SF3B3 occupancy. These results indicate that hnRNPA1 functions to recruit SF3B3, thereby facilitating the AS of MARF1.

MARF1-mediated radioresistance in tumor cells had not been previously reported. In this study, we identified PPP1R10 as a downstream target of MARF1-L. PPP1R10, a regulatory subunit of PP1, primarily functioned by binding to and modulating PP1 activity, thereby participating in various cellular processes [52]. Because efficient DNA repair depended on tightly controlled phosphorylation–dephosphorylation dynamics to coordinate damage signaling and repair complex assembly, PP1 frequently served as a key regulator in these pathways [53, 54]. We demonstrated that MARF1-L promoted the degradation of PPP1R10 in OSCC cells, which in turn enhanced CHK1 phosphorylation and activated HR. Importantly, MARF1-L overexpression partially suppressed the elevation of PPP1R10 expression induced by hnRNPA1 knockdown. Additionally, PPP1R10 overexpression significantly rescued the loss of radiation resistance induced by hnRNPA1 or MARF1-L. Thus, these findings revealed a previously unrecognized regulatory axis in which hnRNPA1 enhanced OSCC radioresistance by promoting MARF1-L–mediated degradation of PPP1R10. Beyond MARF1, transcriptomic profiling further indicated that hnRNPA1 also regulated the AS of a broad set of genes. Therefore, it is essential to investigate whether the AS of these candidated genes also contributed to OSCC radioresistance or influenced other oncogenic processes, such as metastasis, tumor metabolism, chemotherapy resistance, or modulation of the immune microenvironment.

In conclusion, our systematic analysis of splicing factors pinpoints hnRNPA1 as a master oncogenic driver in OSCC. We elucidate a novel mechanism whereby hnRNPA1, in complex with SF3B3, orchestrates a cancer-promoting splicing switch in MARF1, favoring the production of the MARF1-L isoform. This switch instigates a downstream cascade involving PPP1R10 degradation, enhanced CHK1 phosphorylation, and potentiated HR, which collectively underpin acquired radioresistance (Fig. 7H). These findings not only decipher a previously unrecognized AS axis governing DNA repair but also highlight the hnRNPA1-SF3B3-MARF1 pathway as a promising therapeutic vulnerability for overcoming radioresistance in OSCC.

## Supplementary Information

Supplementary Material 1.

Supplementary Material 2.

## Data Availability

The publicly available data are provided in TCGA databases. The data that support the findings of this study are available from the corresponding author upon reasonable request.

## Abbreviations

Abbreviation: Full Term
OSCC: Oral squamous cell carcinoma
AS: Alternative splicing
HR: Homologous recombination
RT: Radiotherapy
hnRNPA1: Heterogeneous nuclear ribonucleoprotein A1
SF3B3: Splicing factor 3B subunit 3
MARF1: MRNA stability factor 1
qPCR: Quantitative real-time PCR
IHC: Immunohistochemistry
WB: Western blot
IF: Immunofluorescence
NHEJ: Non-homologous end joining
FSC: Forward scatter
SSC: Side scatter
siRNAs: Small interfering RNAs
NC: Negative control
MS: Mass spectrometry
RIP: RNA Immunoprecipitation
SPF: Specific pathogen-free
TCGA: The Cancer Genome Atlas
GEO: Gene Expression Omnibus
IR: Irradiation
GSEA: Gene set enrichment analysis
ONT: Oxford Nanopore Technologies
SE: Skipped exons
PSI: Percent Spliced In
PP1: Protein phosphatase 1
CC: Cellular Component
MF: Molecular Function
PPP1R10: Protein phosphatase 1 regulatory subunit 10
PPP1R3G: Protein phosphatase 1 regulatory subunit 3G
GO: Gene Ontology
Co-IP: Co-immunoprecipitation
