# Supplementary material for: hnRNPA1-SF3B3 interaction drives radioresistance in oral squamous cell carcinoma by modulating MARF1 alternative splicing isoforms
Source: J Exp Clin Cancer Res. 2026 Mar 21;45:107. doi: 10.1186/s13046-026-03697-4 (PMC13126795; doi:10.1186/s13046-026-03697-4)
Supplement: Supplementary file 1 — Supplementary Material 1. [file 13046_2026_3697_MOESM1_ESM.docx]

**Supplymentary tables**

**Table S1.** The lists of 134 splicing factors.

| Gene names | | | | | |
| --- | --- | --- | --- | --- | --- |
| ACIN1 | AQR | BCAS2 | BUB3 | BUD31 | C9orf78 |
| CACTIN | CD2BP2 | CDC40 | CDC5L | CHERP | CRNKL1 |
| CTNNBL1 | CWC15 | CXorf56 | DDX23 | DDX35 | DDX41 |
| DDX46 | DDX15 | DHX8 | EFTUD2 | FAM5OA | HNRNPAO |
| HNRNPA1 | HNRNPA2B1 | HNRNPA3 | HNRNPC | HNRNPD | HNRNPDL |
| HNRNPF | HNRNPH1 | HNRNPH3 | HNRNPK | HNRNPL | HNRNPM |
| HNRNPR | HNRNPU | HNRNPUL1 | HSPA4 | HSPA5 | HSPA8 |
| HTATSF1 | HTRA2 | IK | ISY1 | LSM2 | LSM3 |
| LSM4 | LSM6 | LSM7 | LSM8 | MAGOH | MATR3 |
| NAA38 | NFATC2IP | NHP2L1 | NOSIP | PABPN1 | PHF5A |
| PLRG1 | PPIE | PPIG | PPIH | PPIL1 | PPIL2 |
| PPIL3 | PPWD1 | PQBP1 | PRPF18 | PRPF19 | PRPF3 |
| PRPF31 | PRPF4 | PRPF4B | PRPF6 | PRPF8 | PTBP1 |
| PUF6O | PALY | RBM17 | RBM22 | RBM8A | RBMX |
| SART1 | SF3A1 | SF3A2 | SF3A3 | SF3B1 | SF3B14 |
| SF3B2 | SF3B3 | SF3B4 | SLU7 | SMNDC1 | SNRNP200 |
| SNRNP27 | SNRNP40 | SNRNP70 | SNRPA | SNRPA1 | SNRPB |
| SNRPB2 | SNRPC | SNRPDI | SNRPD2 | SNRPD3 | SNRPE |
| SNRPF | SNRPG | SNWV1 | SRP40 | SRP54 | SRRM1 |
| SRRM2 | SRSF12 | SRSF2 | SRSF3 | SRSF4 | SRSF6 |
| SRSF7 | SRSF9 | SYF2 | SYNCRIP | TXNL4A | U2AF1 |
| U2AF2 | U2SURP | USP39 | WBP11 | WDR83 | XAB2 |
| YBX1 | ZMAT2 |  |  |  |  |

**Table S2.** The correlation between hnRNPA1 expression and clinicopathological characteristics was analyzed in microarrays of OSCC tissues.

| Clinicopathological features | hnRNPA1 | | *P* value |
| --- | --- | --- | --- |
|  | Low (n=23) | High (n=37) |  |
| **Gender** |  |  | 0.536 |
| Male | 20(87.0%) | 34(91.9%) |  |
| Female | 3(13.0%) | 3(8.1%) |  |
| **Age** |  |  | 0.194 |
| ≤55 | 16(69.6%) | 31(83.8%) |  |
| >55 | 7(30.4%) | 6(16.2%) |  |
| **Position** |  |  | 0.536 |
| Tongue | 20(87.0%) | 28(75.7%) |  |
| Buccal | 2(8.7%) | 7(18.9%) |  |
| Gingiva | 1(4.3%) | 2(5.4%) |  |
| **Differentiation level** |  |  | 0.465 |
| High | 11(47.8%) | 14(37.8%) |  |
| Moderate | 11(47.8%) | 18(48.6%) |  |
| Low | 1(4.3%) | 5(13.5%) |  |
| **T Stage** |  |  | 0.119 |
| 1 | 0(0.0%) | 7(18.9%) |  |
| 2 | 13(56.5%) | 15(40.5%) |  |
| 3 | 7(30.4%) | 8(21.6%) |  |
| 4 | 3(13.0%) | 7(18.9%) |  |
| **N Stage** |  |  | 0.389 |
| 0 | 15(65.2%) | 18(48.6%) |  |
| 1 | 6(26.1%) | 12(32.4%) |  |
| 2 | 2(8.7%) | 7(18.9%) |  |
| **M Stage** |  |  | 0.818 |
| 0 | 17(73.9%) | 25(67.6%) |  |
| 1 | 4(17.4%) | 9(24.3%) |  |
| 2 | 2(8.7%) | 3(8.1%) |  |

**Table S3.** The correlation between hnRNPA1 expression and clinicopathological characteristics was analyzed in tumor biopsy specimens from OSCC patients undergoing therapeutic radiotherapy.

| Clinicopathological features | hnRNPA1 expression | | *P* value |
| --- | --- | --- | --- |
|  | Low (n = 17) | High (n=12) |  |
| **Gender** |  |  | 0.27 |
| Male | 12 (70.6%) | 6 (50.0%) |  |
| Female | 5 (29.4%) | 6 (50.0%) |  |
| **Smoking** |  |  | 1.00 |
| Yes | 10 (58.8%) | 7 (58.3%) |  |
| No | 7 (41.2%) | 5 (41.7%) |  |
| **Age** |  |  | 0.46 |
| ≤63 | 10 (58.8%) | 5 (41.7%) |  |
| >63 | 7 (41.2%) | 7 (58.3%) |  |
| **HPV status** |  |  | 0.42 |
| Negative | 14 (82.4%) | 8 (66.7%) |  |
| Positive | 3 (17.7%) | 4 (33.3%) |  |
| **Clinical Stage** |  |  | 0.03 |
| III | 3 (17.7%) | 7 (58.3%) |  |
| IV | 14 (82.4%) | 5 (41.7%) |  |
| **Therapeutic efficacy of radiotherapy** |  |  | 0.001 |
| Response (CR+PR) | 2 (11.7%) | 9 (75.0%) |  |
| No-response (SD+PD) | 15 (88.2%) | 3 (25.0%) |  |

**Table S4. Univariate and multivariate Logistic regression models demonstrated the efficacy effects of the A1 expression.**

| **Variable** | **Univariate** | | **Multivariate** | |
| --- | --- | --- | --- | --- |
|  | **HR (95%CI)** | ***P*** | **HR (95%CI)** | ***P*** |
| **Age** |  |  |  |  |
| ≤ 63 years | Ref. |  |  |  |
| > 65 years | 0.67 (0.15, 3.01) | 0.598 |  |  |
| **Gender** |  |  |  |  |
| Male | Ref. |  |  |  |
| Female | 0.60 (0.13, 2.80) | 0.515 |  |  |
| **Smoking status** |  |  |  |  |
| Yes | Ref. |  |  |  |
| No | 0.76 (0.17, 3.49) | 0.728 |  |  |
| **HPV status** |  |  |  |  |
| Negative | Ref. |  |  |  |
| Positive | 0.35 (0.06, 2.00) | 0.238 |  |  |
| **Clinical Stage** |  |  |  |  |
| III | Ref. |  |  |  |
| **IV** | **8.75 (1.53, 50.11)** | **0.015** | 4.96 (0.60, 40.85) | 0.137 |
| **A1 expression** |  |  |  |  |
| Low group | Ref. |  |  |  |
| **High group** | **0.04 (0.01, 0.32)** | **0.002** | **0.04 (0.01, 0.32)** | **0.002** |

**Table S5.** The splicing types and sites of 7 genes related to double-strand break repair, as well as the differences between the two groups (sh-hnRNPA1e vs scramble).

| Gene | Chr | Type | Diff PSI | *P* value |
| --- | --- | --- | --- | --- |
| MARF1 | chr16 | SE | 0.544560847 | 0.026223776 |
| TP53 | chr17 | SE | 0.533971303 | 0.035964036 |
| BRCA1 | chr17 | SE | -0.615488609 | 0.04995005 |
| KAT5 | chr11 | SE | -0.106679842 | 0.018981019 |
| MAD2L2 | chr1 | AF | 0.345651398 | 0.00999001 |
| BRCA1 | chr17 | A5 | 0.542024955 | 0.04995005 |
| TRIP13 | chr5 | AL | 0.420444215 | 0.023476524 |
| XRCC4 | chr5 | AL | -0.240105803 | 0.038961039 |

**Table S6.** The primary antibodies utilized for western blot analysis, immunochemistry, immunofluorescence and Co-IP/RIP.

| Antibody | Company | Catalog Number | Dilution |
| --- | --- | --- | --- |
| hnRNPA1 | Cell Signaling Technology | D21H11 | 1:1000 |
| Phospho-Histone H2A.X (Ser139) | Cell Signaling Technology | 20E3 | 1:1000 |
| SF3B3 | Proteintech | 14577-1-AP | 1:2000 |
| PPP1R10 | Proteintech | 24450-1-AP | 1:1000 |
| Chk1 | Proteintech | 25887-1-AP | 1:1000 |
| Phospho-Chk1 (Ser345) | Cell Signaling Technology | 133D3 | 1:1000 |
| GAPDH | Cell Signaling Technology | 14C10 | 1:1000 |
| hnRNAP1 | Proteintech | 11176-1-AP | 1:1000 |
| Phospho-Histone H2A.X (Ser139) | Cell Signaling Technology | 20E3 | 1:500 |
| Ki-67 | Proteintech | 27309-1-AP | 1:4000 |
| Phospho-Histone H2A.X (Ser139) | Cell Signaling Technology | 20E3 | 1:400 |
| Rad51 | Abcam | ab133534 | 1:200 |
| hnRNPA1 | Cell Signaling Technology | D21H11 | 1:100 |
| SF3B3 | Proteintech | 14577-1-AP | 1:100 |
| PPP1R10 | Proteintech | 24450-1-AP | 1:100 |
| Chk1 | Proteintech | 25887-1-AP | 1:100 |

**Table S7.** The secondary antibodies utilized for western blot and immunofluorescence.

| Antibody | Company | Catalog Number | Dilution |
| --- | --- | --- | --- |
| Anti-rabbit IgG, HRP-linked Antibody | Cell Signaling Technology | 7074 | 1:2000 |
| Anti-mouse IgG, HRP-linked Antibody | Cell Signaling Technology | 7076 | 1:2000 |
| Anti-rabbit IgG (H+L), F(ab')2 Fragment (Alexa Fluor® 594 Conjugate) | Cell Signaling Technology | 8884 | 1:2000 |
| Anti-mouse IgG (H+L), F(ab')2 Fragment (Alexa Fluor® 647 Conjugate) | Cell Signaling Technology | 4410 | 1:2000 |

**Table S8.** The sequences of oligonucleotides and probes used in this study.

| **Name** | **Sequence** |
| --- | --- |
| **siRNA** |  |
| si-NC sense | UUCUCCGAACGUGUCACGUTT |
| si-NC antisense | ACGUGACACGUUCGGAGAATT |
| si-hnRNP A1-1 sense | GAAGAGUUGUGGAACCAAATT |
| si-hnRNP A1-1 antisense | UUUGGUUCCACAACUCUUCTT |
| si-hnRNP A1-2 sense | GGACUGUAUUUGUGACUAATT |
| si-hnRNP A1-2 antisense | UUAGUCACAAAUACAGUCCTT |
| si-SF3B3 -1 sense | CCAGAUAUCCGCUGUCCAATT |
| si-SF3B3 -1 antisense | UUGGACAGCGGAUAUCUGGTT |
| si-SF3B3 -2 sense | CCACGAAAGCUCAGAGAAATT |
| si-SF3B3 -2 antisense | UUUCUCUGAGCUUUCGUGGTT |
| si-PPP1R10 sense | GGAUGAAACUGAACGAGUATT |
| si-PPP1R10 antisense | UACUCGUUCAGUUUCAUCCTT |
| **shRNA** |  |
| shRNA Control | CAACAAGATGAAGAGCACCAA |
| shRNA-1 HNRNPA1  shRNA-2 HNRNPA1 | ACCCGTGAAGGGAGGAAATTT  TGACCATGACTCCGTGGATAA |
| **Plasmid** |  |
| hnRNPA1 | NCBI Reference Sequence: NM_031157.4 |
| PPP1R10 | NCBI Reference Sequence: [NM_002714.4](https://www.ncbi.nlm.nih.gov/nuccore/NM_002714.4) |
| MARF1-L | NCBI Reference Sequence: NM_014647.4(Include exon8) |
| MARF1-S | NCBI Reference Sequence: NM_014647.4(exclude exon8cagtgccacactctgctctatgtttataacctaccagcaaataaggatggcaagagcgtcagcaacaggctcagacgcctgtccgataattgtggtgggaaagtgctgagtatcacaggctgcagtgcaattctccgcttcataaaccaagatagtgcagagcgcgctcagaagcgaatggaaaacgaagatgtctttggtaataggatcattgtgtcatttactccaaaaaatagagaactctgtgaaacaaagagttcaaatgcaattgctgataaagtgaagtctcccaaaaaacttaagaatccaaaattgtgcctcatcaaagatgcaagtgaacaatcttccagtgccaaagccacgcctggaaaagggtcacaggcaaattctggatctgctacaaaaaatacaaatgttaaaagtttacag) |
| **Probes for pull-down assay** |  |
| MARF1 7-1 | 5’-3’:GUAAGUGGGUUUGCGUUAUUUUUGCCAUUUUCCAAUAUUACAUUGUGGAGGCUGAAAGACAGCCCAUAAUAAGGGGGUUGCCAGGCCCAGAUGGGGCUGUUCUUUGUAAGAGGUGGGUUAGAUUUUGAAGUAAGGCUUAGAAACCUUCGGUUCUUCUCACAAAUAUACAGAUAAUUGGUAUGUAUGAAGUUUGCUUUUAUUUAUUUCAAAAUAUCAUAUGAAAUUGACUUGUAGAUUUACCUUCCCAACUUGCCAGUGUCUUCAAAGCUAGCAGUGUUUUGUGUCAGGUGGAUAGAAAUAACGGAUUAAAAGUCAUGAUUCUUUUUGCUUCCAAGUAUUAUGGGUAGAGAAUAUGGCGAGGGUUCUCACAUACUUGUGUGGUUGGCACCAAAUAAGUGGAGUCAAUUGUGCAUUUUUUUCGGUAUUUAAUCCUUAAGCUUCAGCGGGCCUCUGAGUCUGUGCUUUCUAUUGUGCAUUCCCUGUGGUGCAUGGACACAAUUAGCAGGAGUGGUUAUUGCUCCACCUGUGUGUAGCCUGUUUAUGCCAUAUACUGGGAGGAGGGUGUGAGCACAUUUCCAGGACUAGCAUUUCUGAUAGACUUGACUUGGGAUAAGAAUGUUUAUUGCAGUGCUGGGCUGUGUUUAAGCCGCUGGCGAAUAUGUGUGUAACUCUGAAAGAAUUAAUGAUAAUGGAGAAGGAGCCAUGGUGUGGGUGAUCCAGGAGAGUGGGCAAUCCCAUACGUUAAAUGAAAGUCUUCACAUAUAAUUUAAUAACUGAGAGUUACAGAAGUACGUUAGACAUGAUCUCAUGCAAAGUCUUCAUUUAAGGGAGAAAGAAAUCAGCUCUUAGAAAAAGUAAACGUGUCAUUGGUGAUUGCAGCAGUUAAGAGAGUGGGCCCCGGGUAUCCUGAUUCCGGCACUUGCCUGCUAGUCACCUGUGUCCCCGAUACCCAACAAGCCCCCUGCGGUCUGGCUUAAGUUGAAACUCAUGCUAUGGACAGUUGAGAGUUGUGGAUACCAGAGGCUUACCCAGGGAGUAAGCAUAUACAGGCCUUACCUUAAUCUUUUGAGGCUCUUUCCUAACUCACCUUUGUCAUUAUUUGUUUUUAGUCACUUGGCUGUAGUUACAUUUCUUCCCUUCUGCUUCUGUUUGACGGUCACUUUUCUAACUUUGAAGGCCUUAUGUAAAUACGGUGUUUUUUCCUGGGAGGUGAAGGUUGCAGUGAGCUGAGAUCGCGCCACUGCACUCCAGCCUGGUCGACAGAGCGAGACUCUGUUUCAAAAAAAAAAAAAAAAGUAAAUGUAGUGUUUUAGGUAAGAACUUGCCCACUUUGGAGAUUCUUAUUCCUUAUGGUUAAAAAAACCAAAACAAUGUUUUGAUAAUUCAAACCAUUUAGUUUUGCUGGAUGUUUUUUCCCAUCCACUAGAGUGAUAAUUUAAGAUACAUUUCUUUUUAUGUUCUUUAGUCUGUUCCUUUAAUUUUAAUAAAUUAAACAUACAUUGGUACUGCCUGGGUUUUGAGUCCCAGGUGUAAUAGGAAAAGUGUAGAUUUUGUUGAUAAUUGGUCAGUACCCCUCAAAUAAAAAGAAAAGCCUCUGAUUAGAAAAGUAUGGUGUGGGCCGAGUGAUGUGGCUCACGCCUGCAAUCCCAGUACUUUGGGAAGCUGAGGUGGGCAGAUCACCAAUCACCUAAGGUCAGGAGUUCGAGACCAGCCUGGCCGAUGUGGCGAAACCCCAUCUCUACUAAAAAUACAAAAAUUAGCCAGGUGUGGUGGCGGGCACUGGUAGUCCUAGCUGCUUGGGAGGCUGAGACAGGAGAAUCACUUGAACCUGAAAGUGGAGGUUGCAGUAAGCCGAGAUCACACCACUGCACUCCAGCCUGGGUGACAGAGUAGACUGUGUCUCAAAAAAUCUAAAAAAAAAAGAAAGAAAAGAAUAGUAUGGUAUGAUCAUUGUAAAAACAAACAAAACACAAUAUUUAGAAAUGUGGAAAGUGAAAACCCCACAAUUCUACCCUCCAGAAGUAACCACUGUUAAUAUUUUGGUAUUGCCCUCCAGAUUUAAAAAAUGUACGUUUAAAAAAAAAUGUGUCACAUAUACAGUGUUCUGUGGCUUUUGGAAAAAAACUAUGUCAGAGCUUGGAUGUCUUUUCAUGUUAAUAUAUAUUAAUAUAAAUCUGCCACAUUUUAAAAACCAGAACUUUGGUUUGAAAAGUACUUGUAUGGCUGGUCUUUAUUUAACCAGUCUACCUGUUAGUGGACAUUUUG |
| MARF1 7-2 | 5’-3’:UUUAUACCCAGUUUUGUGUUUUGUUUUGUUUUUUACCAUUUCCAGCAAGGUAAUUUAUAUCUUUAGGCUCCUGGGCUGACAUACCAUAGGGUAAUUUCCAAGGCAUAAGAGUUCUGGGUUAGAACUUUACUAGCAAGAUCCAUGUCUUCCUCCAGGAAGGUGGUACUAAUUUACACUCAUAAGAGUGUCUGUUUUAAAUGUUUGCCAGGCCAGUAAGUGAAAAACAGCAUCAUCUUAUAAUUGACUUUUAAUCUUUAACACAGUGCAAAAUAUCCUUUUAUGAUUAUCGUUGUUUGCUUUUUAUUUCUUCUGUAAAUUGUUCACAUCCUUUGCCUGUUGUUAGUGAUUUCUCUUAUGGUUUCUGGAUUUAACCCUUUAUAUAAUUAUCUUAAGUUUUCUCCUGGUGCUUUAAUGGUUCUGCUUUUUAAAAUAUUAUUCUUAUUUAAAAAAAAUUUUUUUGAGACUCUGUCACCCAGGCUAGAGUGCAGUGGCGUGAUCUCCGCUUACUGCAGACUCCACCUGCCGGGUUCAAUUGAUUCUCCUUCCUCAGCCUCCCGAGUAGCUGGGAUUACAGGCGCCCGCCACCACGCCUGGCUAAUUUUGUAUUUUUAGUAGAGAGGGGGUUUCACUGUGUUGGCCAGGCUUGCCUCGAACUUCUGACCUCAGGUGAUCACCCGCUUUGGCCUCGCAAAGUGCUGGGAUUACAGGCGUGAGCCACCGUGCCCAGCCUCUGGCUAGUUUUUUGUAGACGAGGUUUCACCAUGUUGGCCAGGCUGGUCUUGAACUCCUGACCUCAAGUGAUCCACCUGCCCCAGCAUCCCUAAGUACUGGGAUUAGAGGAGUGAGCCACAAUGCCUGGCAAUUUUUAAAAAUUUUUAAUAGAGAUGGGGUCUCACUGUGUUGCUCAGGCUGGUUUUGAACUCCUGGGCUCAAGUGAUCCCCCCGCCUCGGCCUCCCAAAGUGCUGGGAUUACAGGCAUGAGCCACCAUGCCUGGCCAGUUCGUUCGUUCGUUCGUUUGUUCGUUCUUUCUCUCUCUCUCUCUCUUUUCUUUCUUUUUCUCCAGUUCUACUUCCUUCUUUCUUUCUUUCUUUUUUUUUUUUUUUUCACAGAAUCUCGCUCUGUCGCCCAGGCUGGAGUACAGUGGUGCCAUCUCAGCUCACUGCAACCUCCGCCUCCUGGGUUCAGGCAAAUUAUUCUGCCUCAGCCUCUGGAGUAGCUGGGAUUACAGGCGUCUGCCACUUUGCUCAGCUAACUUUUUUUGUAUUUUUAGUAGAGAUGGGGUUUCACCAUGUUGGUCAGGCUGGUCGCCAACUCUUGACCUCAGGUGAUCCACCUGCCUUGGCCUCCCAGAGUGUUGCGAUGACAGGCGUGAGCCACUGUGCCAGGCCAAGUUCUACUUCUUAAUACAUAAAUUUCAACUUAUCUGGAAGAAUUAUGACUACCCACUGCCAAAUAAAACUUCCAGCCUAACUACUGGCAAUCUGCUGUUGAAGCUGAACAGGCUCUAAAUUGUUGGGUUUUAAAAAAAUUUUUGUUUACUUAAGCCUAUAGCUCCUAGUAUUCCCAGGCAUUCUCCUAUGCAAAAACCAACCAGGACUGACCCUGCUCACCUUCUGGCUCUAUAAGUUAUUAUUCAGCAGACCUGCAGAAUAAAUAGACUUUUAAAAAACAACUUCGUUGAGAUAUGAUUUACAUAUUACAAAAUUCAGCUCUUUUAAGUGUACAAUAAUUUUUAGUAAAUUGAGUUGUACAAUUUUAGAAUAUUUUUGUCACCUCAGUAAAUCUAUUAUGCUAAUUUAUAAUUAAUCCCCUUCCCCACUCCCAGGCACUACUAAUCUUUCUGUCUCUGUAGAUUUGUAUUUUCUGGAUGCUUUAUAGAAAUGGAAUCAUAUAGUAUACAGACCUCUGUGCCUAGCAUAUUCUUUUUUAUUUUUAUUUUUUGAGACAGUGUCUCACUCUGUCACUCAGAGUGGAGUGCAGUGGCACAGUCACUCCAGCCUCAAUCUUCCUGGGCUCAGGUAAUCCUCCCACCUGAGUAGCUGGGACUGUAGGCAUGCACCACCAUGCCCAGCUAAAGCAUAUUCUUUAAAACAAUAUUCAGUUACUUUGUCAACACUAAAUAAUUUACCCUCUCCCAUCUAAAUCACUGUCAUCUUCUCUUCAAAGUUGUUAACUGAAGUUUGGAGGUCACCCAUUGUUCUUAUUUUGAAAUUGAAUGUGUGCCCUUGAAUUAACCUGACGUAACACUUCUUUCCUCUGUAUUAG |
| MARF1 8-1 | 5’-3’:GUAAUUUUGAUACCUCUUGCUUUCUGAAGUUUAUGGUAGGUUUGGUUUGUUUCUGUGUUUUACGUGCCCGCUUGCUUUUGGCGUGUCCCUUUUUGAUUUC |
| MARF1 8-2 | 5’-3’:AGUGUUUGAUGAUACUCAAAGUCAAUCGUUUUCUGUAAAGGAUCUAACACAUCUUGGGUACUUAAAAUUUAAACCCCACUGUGCUUGUGUCUUUGAAG |
| MARF1 8-1 Mut | 5’-3’:GUAAUUUUGAUACCUCUUGCUUUCUGAAGUUUAUGG---GUUUGGUUUGUUUCUGUGUUUUACGUGCCCGCUUGCUUUUGGCGUGUCCCUUUUUGAUUUC |

**Table S9:** Primers used in the present study.

| **Name** | **Sequence 5’-3’** |
| --- | --- |
| GAPDH Forward | GTCTCCTCTGACTTCAACAGCG |
| GAPDH Reverse | ACCACCCTGTTGCTGTAGCCAA |
| hnRNPA1 Forward | CTTTGGCGGTGGTAGTGGAAG |
| hnRNPA1 Reverse | GAACCGCCATAGCCACCTTG |
| PPP1R10 Forward | CTGGGCTTTCTGGATGCTCT |
| PPP1R10 Reverse | GAAGGTTTGGCTGTGCTTGG |
| MARF1 Forward | GAAGAGTTCATTTCCGACTTGCC |
| MARF1 Reverse | TGCTGGTGCTCACTGTTTCTA |
| SLAMF7 Forward | TGCCTCACCCTCATCTATATCCT |
| SLAMF7 Reverse | CAGGTGCCATTCTTATTGCTCTG |
| MICA Forward | CTTGACAGGGAACGGAAAGGA |
| MICA Reverse | GTCTTGGTCTTCATGGCATCTTC |
| PTPMT1 Forward | GAGTACGAGACGAGGTTCCTG |
| PTPMT1 Reverse | ATCTTGGCGATGGCTCTTACA |
| EIF4A2 Forward | GGATTGATGTGCAACAAGTGTCT |
| EIF4A2 Reverse | GCATCTCCTCCACTGTAGTATTGT |
| VARS2 Forward | GGTAGCAGCGGAACTGACA |
| VARS2 Reverse | CCCACCCAGAACAGCAGAA |
| PPP1R3G Forward | CAGCTACGTGAAGCTGAGCA |
| PPP1R3G Reverse | GCACAGTTGGCGATCTTGGT |
