# Supplementary material for: hnRNPA1-SF3B3 interaction drives radioresistance in oral squamous cell carcinoma by modulating MARF1 alternative splicing isoforms
Source: J Exp Clin Cancer Res. 2026 Mar 21;45:107. doi: 10.1186/s13046-026-03697-4 (PMC13126795; doi:10.1186/s13046-026-03697-4)
Supplement: Supplementary file 2 — Supplementary Material 2. [file 13046_2026_3697_MOESM2_ESM.docx]

**Supplymentary figures**

**
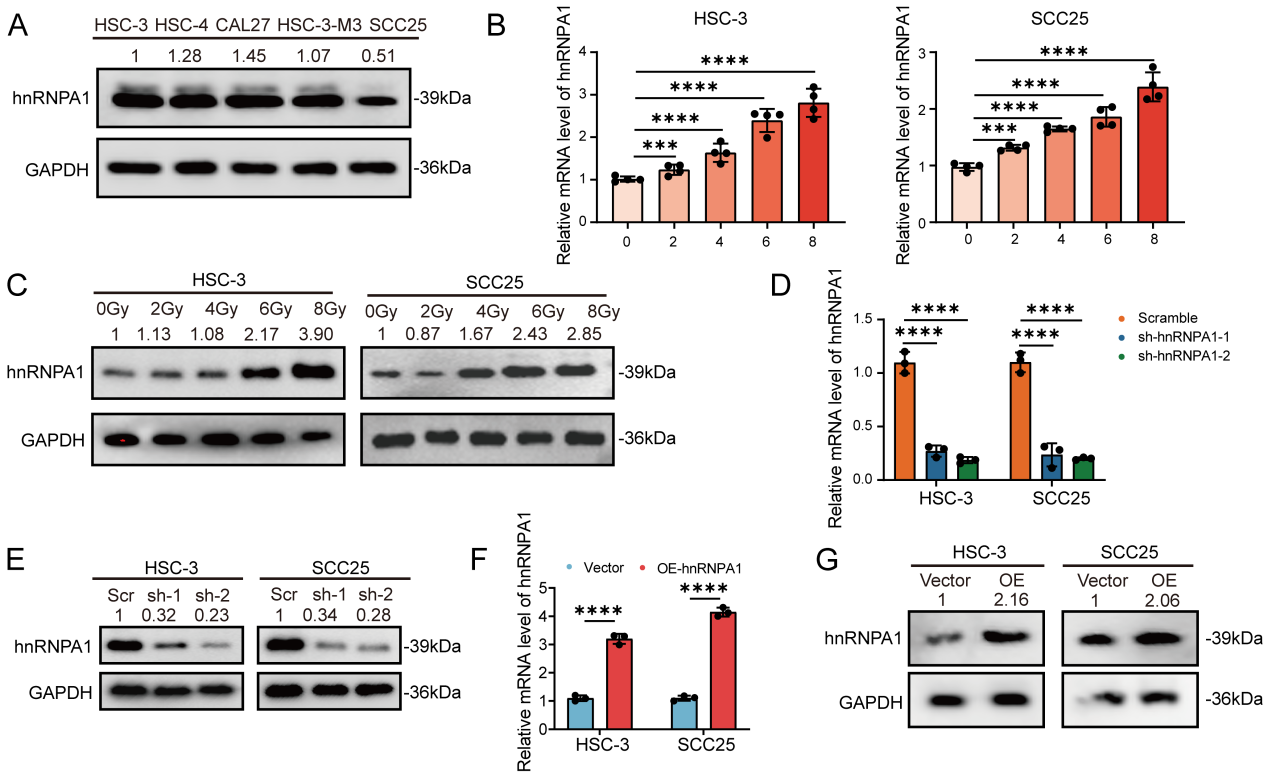
**

**Figure S1. Assessment of hnRNPA1 levels in OSCC cells after radiotherapy and validation of hnRNPA1-overexpressing and knockdown OSCC cell lines.** (A) Western blot analysis of hnRNPA1 protein expression in five human OSCC cell lines; (B) qPCR analysis of hnRNPA1 mRNA expression in OSCC cells exposed to different radiation doses (0, 2, 4, 6, and 8 Gy); (C)Western blot analysis of hnRNPA1 protein expression in OSCC cells under the same radiationconditions; (D) qPCR analysis of hnRNPA1 mRNA expression in OSCC cell lines with stable hnRNPA1 knockdown; (E) Western blot analysis confirming hnRNPA1 protein knockdown in OSCC cell lines; (F) qPCR analysis of hnRNPA1 mRNA expression in OSCC cell lines with stable hnRNPA1 overexpression; (G) Western blot analysis confirming hnRNPA1 protein overexpression in OSCC cell lines. * p < 0.05; ** p < 0.01; *** p < 0.001; **** p < 0.0001; ns, not significant.


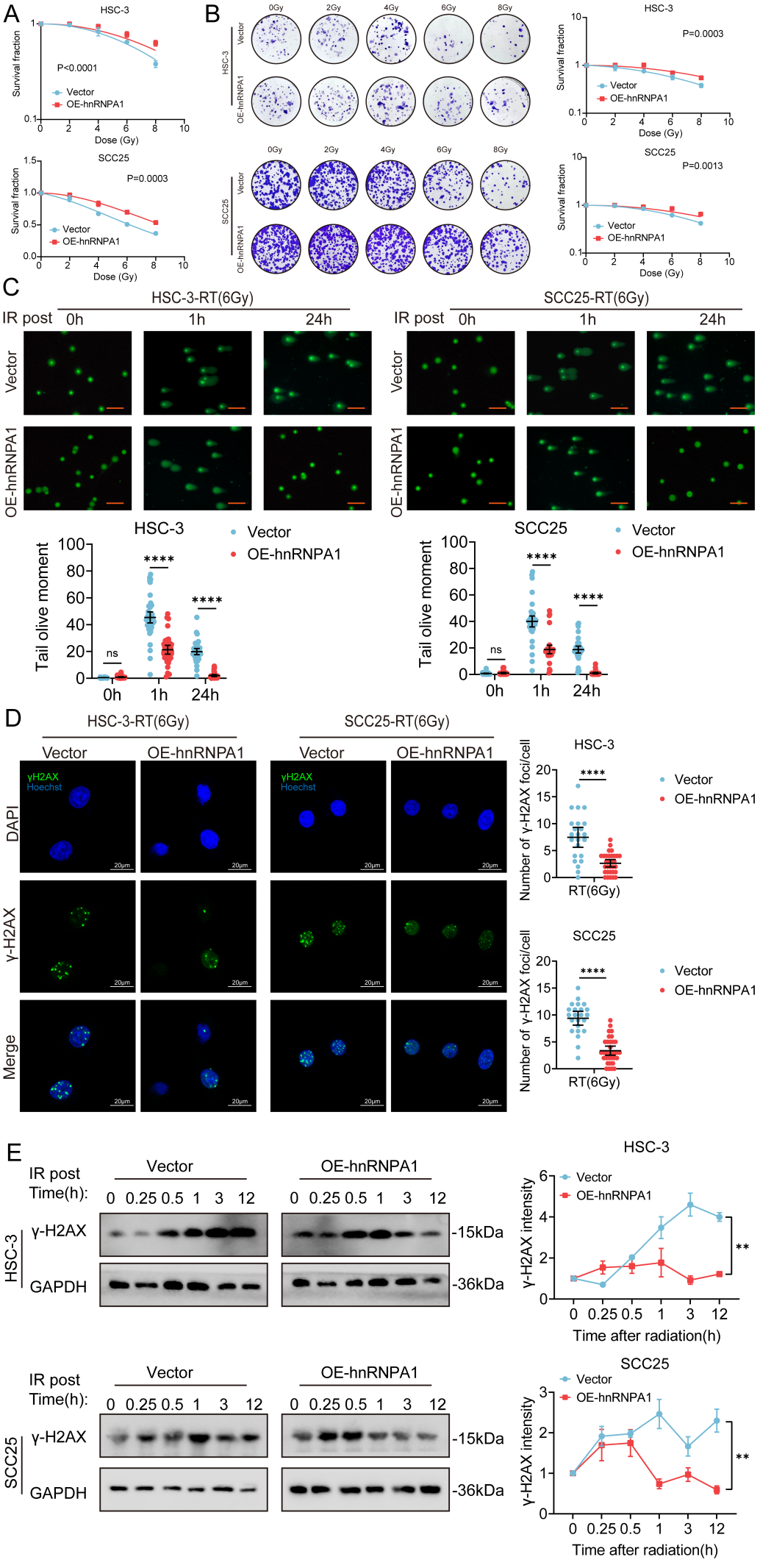


**FigureS2. Overexpression of hnRNPA1 promoted radioresistance in OSCC cells in vitro.**(A) CCK-8 assay assessing the survival rates of OSCC cells in the OE-hnRNPA1 and control groups following 96 hours of treatment with different radiation doses (0, 2, 4, 6, and 8 Gy); (B) Clonogenic assays evaluating the clonogenic ability of OSCC cells in the OE-hnRNPA1 and control groups following 2 weeks of treatment with different radiation doses (0, 2, 4, 6, 8 Gy); (C) Comet assays detecting DNA damage in OSCC cells from the OE-hnRNPA1 and control groups at 1 and 24 hours post-6Gy IR, Scale bar, 20µm; (D-E) Immunofluorescence assays assessing γ-H2AX foci formation in OSCC cells from the OE-hnRNPA1 and control groups at 12 hours post-6Gy IR, Scale bar, 20µm; (F-G) Western blot analysis of γ-H2AX protein levels in OSCC cells from the OE-hnRNPA1 and control groups at 0.25, 0.5, 1, 3, and 12 hours post-6Gy IR. * p < 0.05; ** p < 0.01; *** p < 0.001; **** p < 0.0001; ns, not significant.


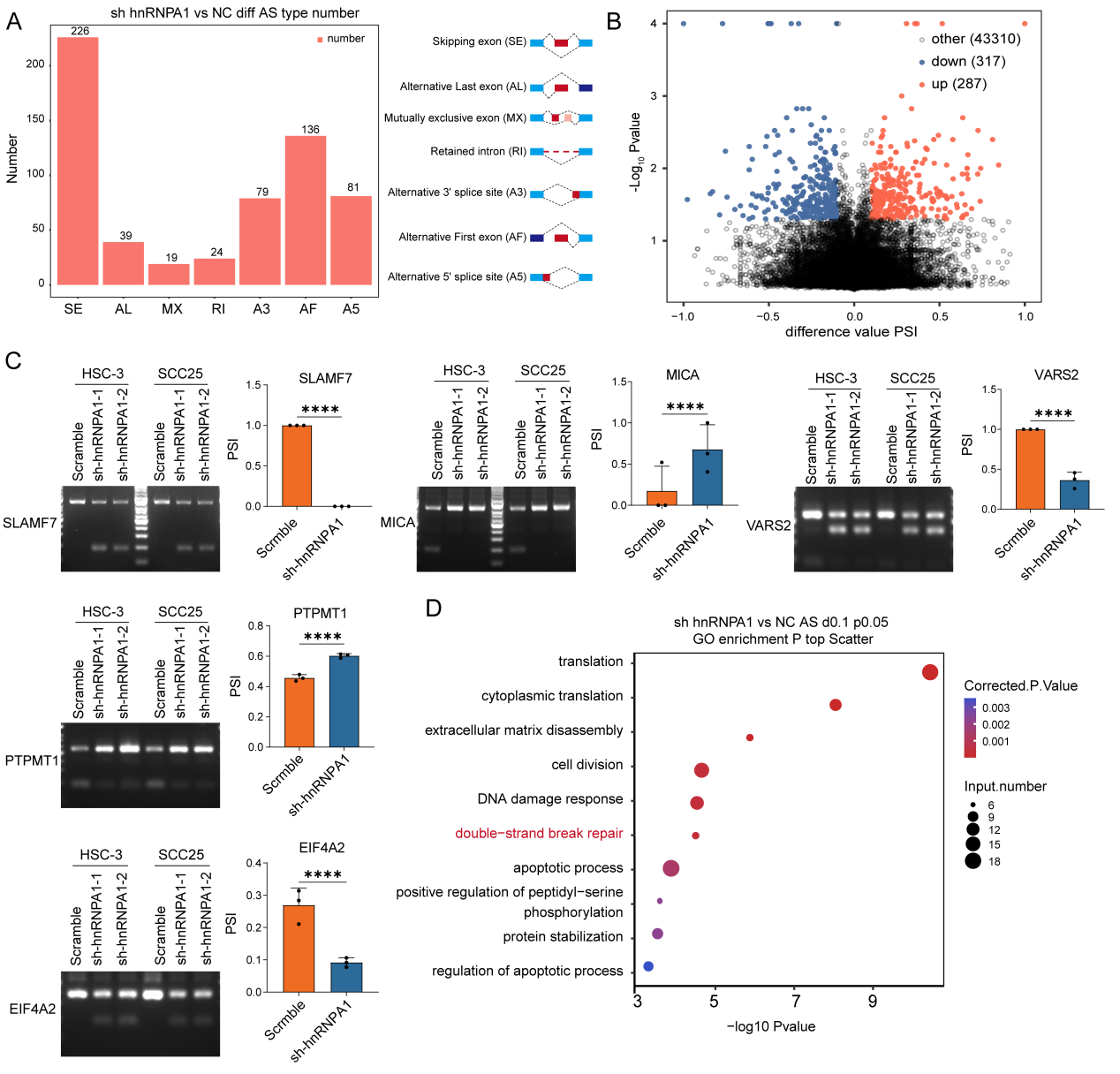


**Figure S3. hnRNPA1 regulated AS and GO enrichment analysis of splicing-altered genes.**(A) Quantitative analysis of seven types AS eventswas performed in HSC-3 cells stably transfected with sh-hnRNPA1 lentivirus; (B) Volcano plot showing the distribution of AS events with increased or decreased difference values of PSI (Ψ) between the sh-hnRNPA1 and control groups; (C) Verification of the top five skipped exon (SE) events in hnRNPA1-knockdown OSCC cells by agarose gel electrophoresis of qPCR products and its RNA-seq PSI statistics; (D) GO enrichment analysis of splicing-altered genes between sh-hnRNPA1 and control groups. * p < 0.05; ** p < 0.01; *** p < 0.001; **** p < 0.0001; ns, not significant.


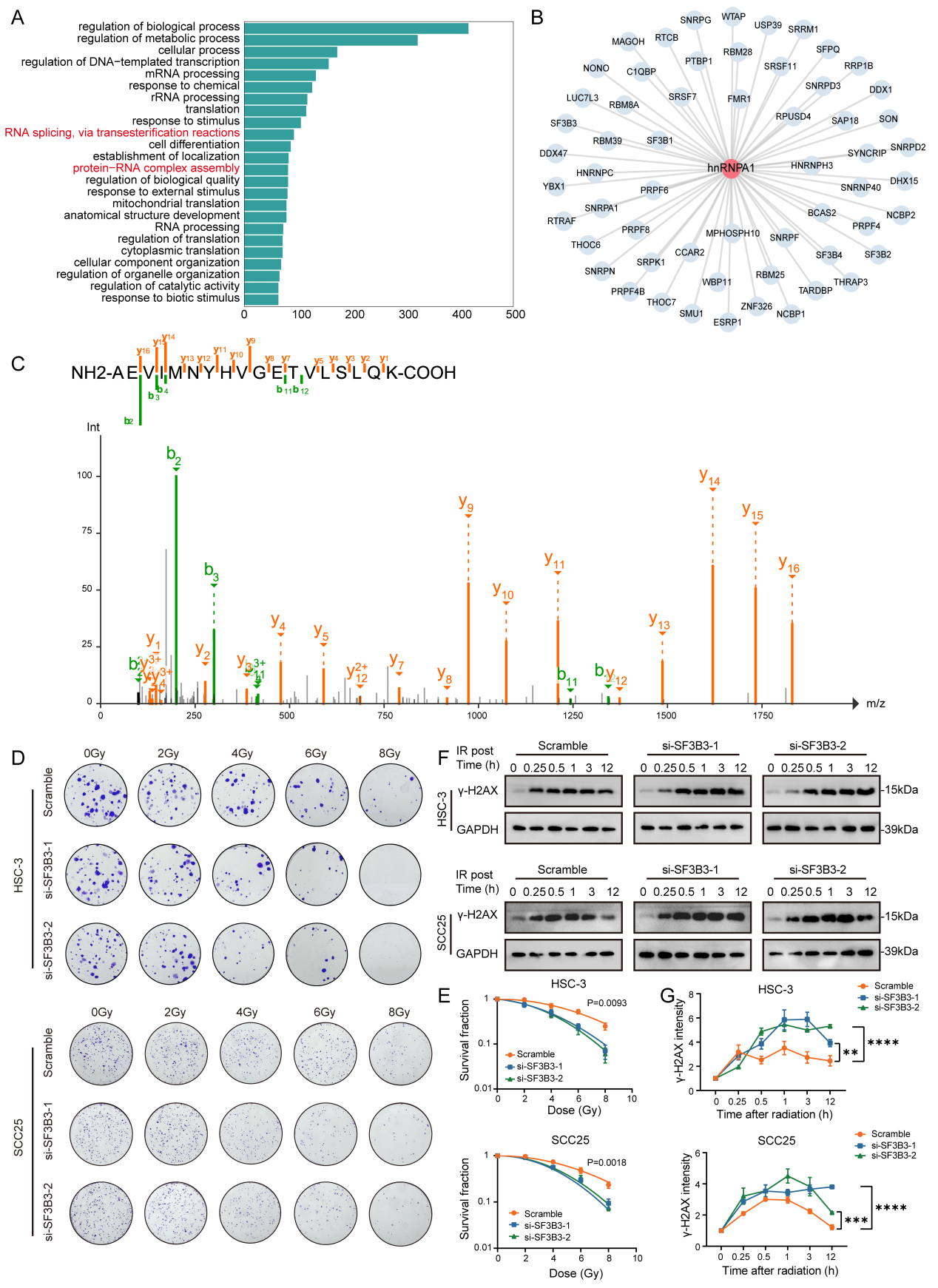


**Figure S4. hnRNPA1 Associated with SF3B3 to Modulate MARF1 Splicing in OSCC.**

1. GO enrichment analysis of hnRNPA1-interacting proteins in HSC-3 cells (bar plot); (B) Mass spectrometry (MS) identification of hnRNPA1-associated AS proteins in HSC-3 cells; (C) Representative peptide of SF3B3by mass spectrometry; (D) clonogenic assays and (E)statistical analysis of the clonogenic ability of OSCC cells in the si-SF3B3 and control groups following 2 weeks of treatment with different radiation doses (0, 2, 4, 6, 8 Gy); (F) Western blot and (G)statistical analysis of γ-H2AX protein levels in OSCC cells from the si-SF3B3 and control groups at 0.25, 0.5, 1, 3, and 12 hours post-6Gy IR. * p < 0.05; ** p < 0.01; *** p < 0.001; **** p < 0.0001; ns, not significant.


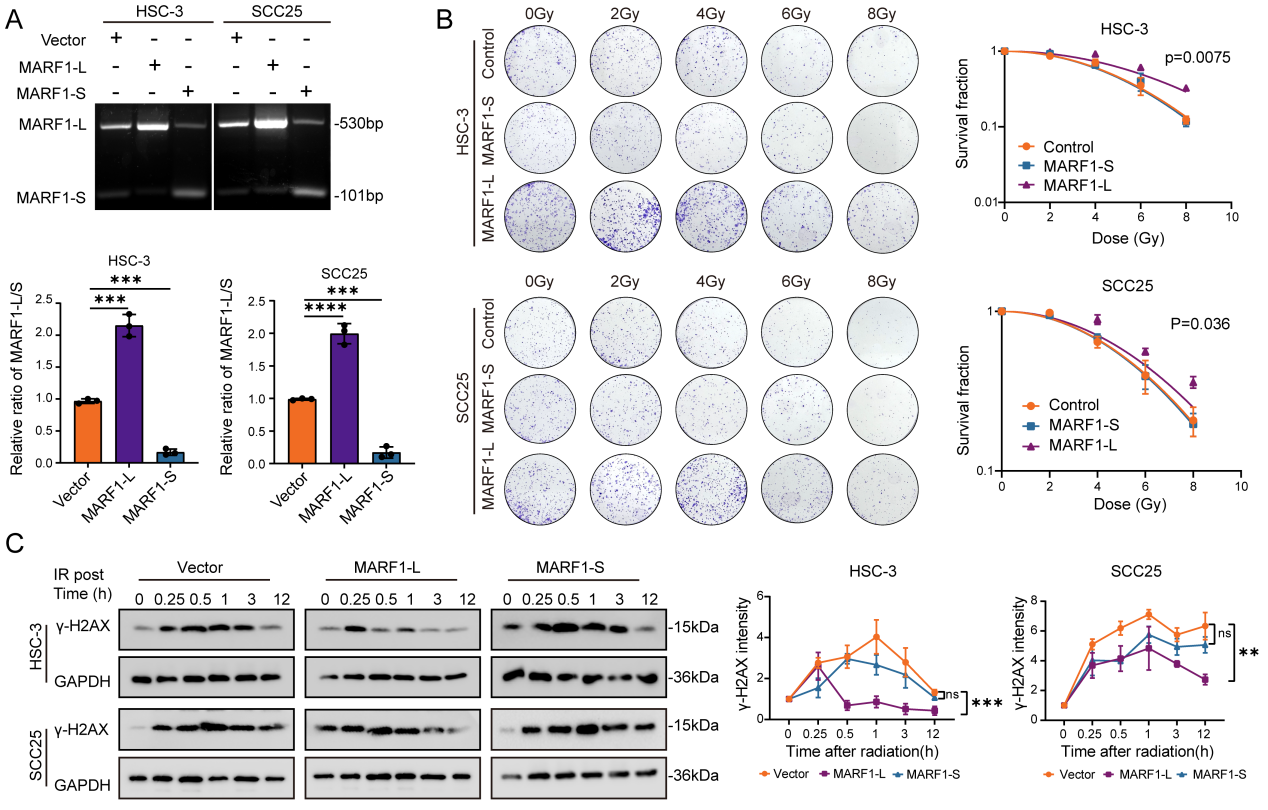


**Figure S5. MARF1-L Mediated Radioresistance in OSCC, Whereas MARF1-S Did Not.** (A) Representative gel electrophoresis image and statistical analysis of the MARF1-L/MARF1-S ratio in OSCC cells overexpression MARF1-L or MARF1-S assessed by qPCR; (B) Clonogenic assays and statistical analysis of the clonogenic capacity of OSCC cells overexpressioning MARF1-Lor MARF1-S following exposure to different radiation doses (0, 2, 4, 6 and 8 Gy); (C) Western blot analysis of γ-H2AX protein levels in OSCC cells from the overexpression MARF1-L or MARF1-S and in control cells at 0.25, 0.5, 1, 3, and 12 hours after 6 Gy IR. * p < 0.05; ** p < 0.01; *** p < 0.001; **** p < 0.0001; ns, not significant.


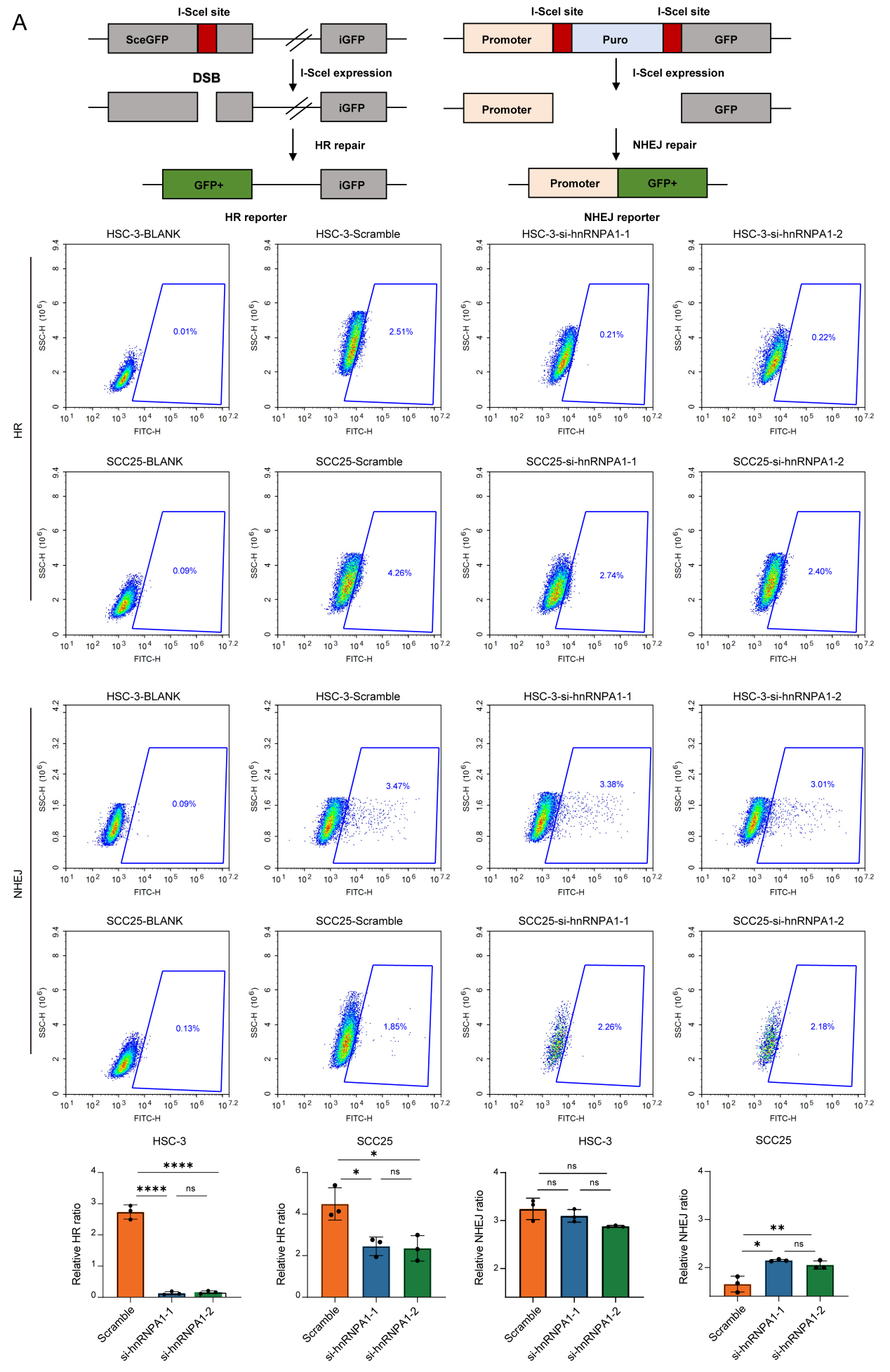


**Figure S6. hnRNPA1 Promotes Radiaresistance in OSCC by Enhancing HR.** (A) Schematic of the DR-GFP/NHEJ-GFP reporter system. HR and NHEJ repair efficiency in the scramble si-hnRNPA1 HSC-3 and SCC25 cells. * p < 0.05; ** p < 0.01; *** p < 0.001; **** p < 0.0001; ns, not significant.


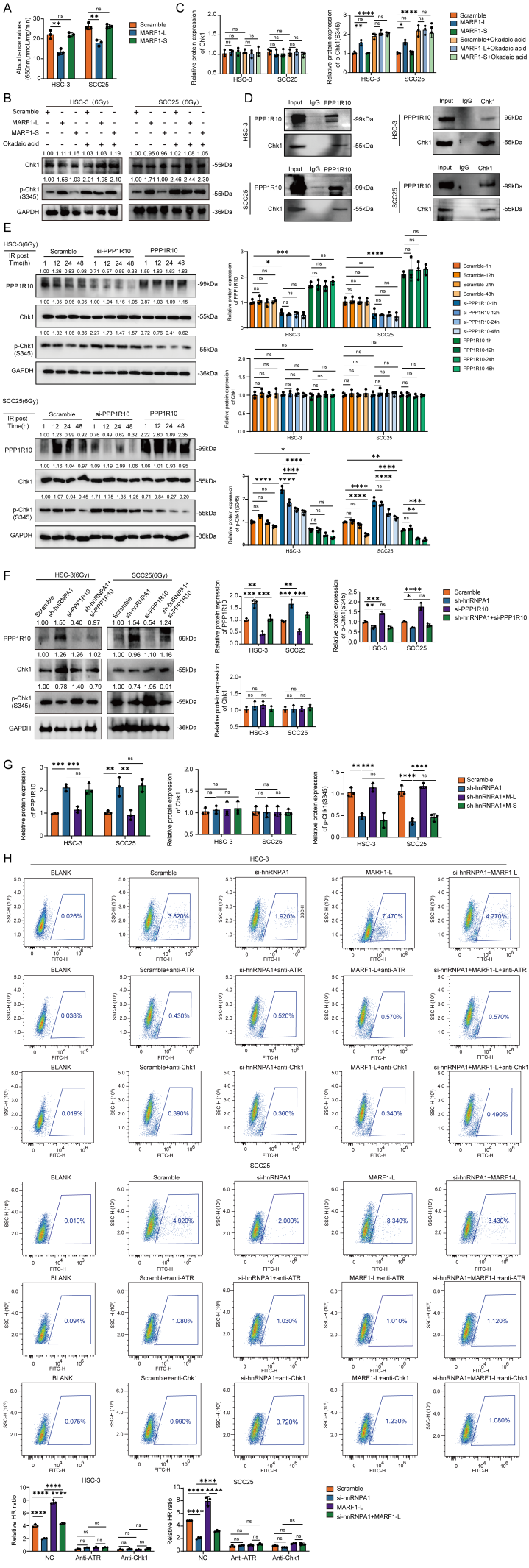


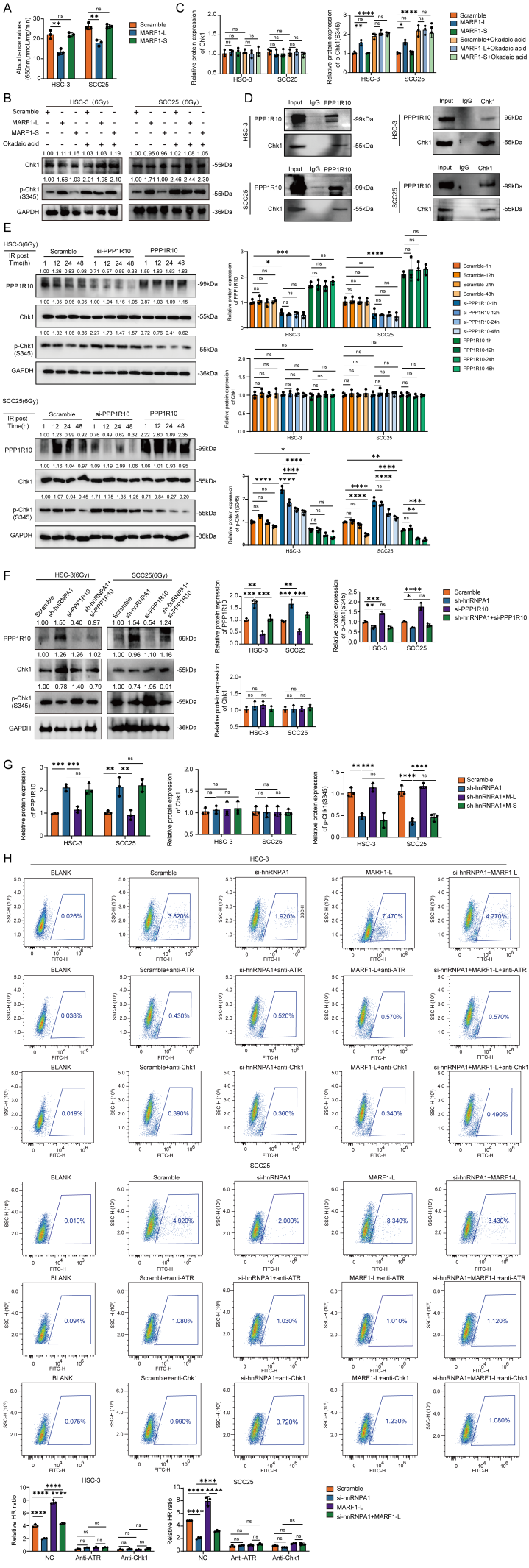


**Figure S7. hnRNPA1 promotes DNA repair by facilitating MARF1-L–dependent degradation of PPP1R10 mRNA to activate CHK1. ​**(A)​ PP1 phosphatase activity measured by a colorimetric assay in HSC-3 and SCC25 OSCC cells overexpressing the indicated MARF1-L or MARF1-S isoforms；(B-C)​ Western blot analysis (B) and quantification (C) of phospho-Chk1 (Ser345) and total Chk1 in OSCC cells under the indicated conditions. The increase in Chk1 phosphorylation induced by MARF1-L overexpression is abolished by treatment with the PP1 inhibitor okadaic acid (OA, 25 nM, 24 h); (D) Co-IP assay confirming the interaction between endogenous PPP1R10 and CHK1 proteins in OSCC cells. IgG was used as a negative control; (E) Western blot analysis of phospho-Chk1 (Ser345) and total Chk1 in HSC-3 cells following PPP1R10 knockdown or overexpression;(F)​Western blot analysis of phospho-Chk1 (Ser345) and total Chk1 in OSCC cells after knockdown of hnRNPA1 or PPP1R10;(G)​Quantification of PPP1R10 and phospho-Chk1 (Ser345) protein levels in hnRNPA1-deficient OSCC cells with or without reconstitution of MARF1-L;(H) HR repair efficiency measured by DR-GFP reporter assay in OSCC cells under the indicated genetic and pharmacological treatments (ATRi, 2 µM; CHK1i, 1 µM; for 24 h). * p < 0.05; ** p < 0.01; *** p < 0.001; **** p < 0.0001; ns, not significant.
